# From indicators to governance: a pressure–condition–response framework reveals nonlinear ecological responses to multiple stressors in an agricultural-urban basin

**DOI:** 10.1007/s10661-026-15608-3

**Published:** 2026-07-02

**Authors:** Karling Fernanda Schuster, Raquel de Brito, José Francisco Gonçalves-Júnior, Camila Campos, Cassia Alves Lima-Rezende, Renan de Souza Rezende

**Affiliations:** 1 Graduate Program in Environmental Sciences, Community University of Chapecó Region-Unochapecó, Chapecó, Santa Catarina 89809-000 Brazil; 2 AquaRiparia-Limnology Laboratory, Department of Ecology, University of Brasília-UnB, Campus Darcy Ribeiro, Asa Norte, Brasília, DF 70910-900 Brazil

**Keywords:** Ecological thresholds, TITAN, Random forest, Water security, Subtropical streams

## Abstract

Understanding how multiple interacting stressors modulate aquatic community dynamics is fundamental to forecasting ecosystem trajectories under accelerating global change. In subtropical basins, the concurrent influences of thermal regimes, ionic composition, and habitat structure generate nonlinear and often synergistic ecological responses that remain unresolved. In this study, we integrated community-level indicators with multivariate and threshold-detection analyses to identify principal environmental drivers and associated biological breakpoints across anthropogenic gradients. An adapted multicriteria index was employed to translate these ecological patterns into operational assessments applicable to regional water governance. Water temperature emerged as the dominant environmental filter regulating both macroinvertebrate and phytoplankton assemblages, exerting a stronger effect than ionic or geomorphological control. Community transitions were characterized by the replacement of thermosensitive taxa with tolerant and generalist groups, concomitant with pronounced reductions in taxonomic richness. Nonlinear responses included a peak in diversity at intermediate ionic concentrations and a critical dissolved oxygen window, beyond which biological integrity declined abruptly. Threshold analyses revealed consistent limits associated with temperature, conductivity, and substrate composition, whereas the multicriteria index effectively detected moderate disturbance but exhibited reduced sensitivity at the extremes, a compression pattern typical of linear scoring systems. Collectively, these results demonstrate that ecological vulnerability in subtropical streams arises from the interaction among thermal stress, oxygen limitation, and habitat simplification, rather than from isolated environmental gradients. By integrating multivariate and threshold-based diagnostics, this framework offers a scalable, mechanism-informed tool to anticipate ecological regime shifts and strengthen adaptive water governance under intensifying climatic and anthropogenic pressures.

**Supplementary Information:**

The online version contains supplementary material available at 10.1007/s10661-026-15608-3.

## Introduction

Streams and small rivers sustain a disproportionate portion of global biodiversity and provide essential ecosystem services, including water supply, nutrient regulation, and fisheries (Allan, 2007). However, these ecosystems are increasingly threatened by climate warming, which has emerged as a major ecological driver in freshwater environments (Huang et al., 2025; Su et al., 2025). Rising water temperatures directly constrain aquatic organisms through physiological stress while simultaneously altering key physicochemical processes, including oxygen solubility, metabolic activity, nutrient cycling, and eutrophication dynamics (Pérez et al., 2023; Tassone et al., 2025). Because temperature regulates both organismal performance and ecosystem functioning, warming can amplify the effects of additional environmental stressors and generate nonlinear ecological responses. The consequences of these thermal alterations are already evident worldwide, including heatwave-induced mass mortality events, biodiversity loss, and shifts in aquatic community composition (Bonacina et al., 2023; Johnson et al., 2024).

Recent advances in environmental monitoring have revealed that water quality assessments are highly sensitive to spatial and temporal resolutions (Elfferich et al., 2024; Rezende et al., 2014). Coarse sampling intervals can distort temporal variability and obscure temperature-sensitive dynamics in aquatic ecosystems, masking short-lived yet ecologically significant events such as nocturnal hypoxia and nutrient pulses (Bieroza et al., 2023; Elfferich et al., 2024; Huang et al., 2025). These events are among the most common episodic processes intensified by temperature, as higher temperatures reduce oxygen solubility, accelerate microbial metabolism, and increase nutrient turnover and oxygen demand (Ballarin et al., 2023). Consequently, failing to capture these transient fluctuations may underestimate their influence on ecosystem resilience and water security (Han et al., 2025; Sultana et al., 2020).

Within bioassessment frameworks, macroinvertebrates and phytoplankton remain cornerstone indicators of ecological integrity owing to their responsiveness to physical, chemical, and hydromorphological gradients, as well as their ability to integrate processes across spatial and temporal scales (Bou et al., 2025; Liu et al., 2024; Mureithi et al., 2025; Zhang et al., 2024). Classical macroinvertebrate-based biomonitoring indices, such as the Biological Monitoring Working Party (BMWP), the Ephemeroptera–Plecoptera–Trichoptera (EPT) richness metric, and multimetric integrity indices, are widely used to assess ecological condition in freshwater ecosystems (Mendoza et al., 2025; Ruaro et al., 2024). However, these approaches often require regional calibration to reduce biogeographical bias and enhance predictive accuracy, particularly in tropical and subtropical systems (Hora Revilla et al., 2025; Prakoso et al., 2024). Recent syntheses have further emphasized the importance of adapting biomonitoring metrics to capture nonlinear ecological responses and improve diagnostic precision across both low- and high-impact conditions (Esmaeili Ofogh et al., 2024; Reyes-Celis et al., 2025; Ruaro et al., 2024; Simaika et al., 2024).

To address these challenges, contemporary analytical approaches increasingly integrate machine learning and threshold-detection techniques (Rodeles et al., 2025; Taylor et al., 2023). Such integration provides a powerful framework for converting complex ecological signals into management-relevant diagnostics that are suitable for tropical and subtropical contexts (Ruaro et al., 2024; Simaika et al., 2024). Whereas classical biomonitoring indices are useful for detecting ecological impairment, integrated assessment frameworks have increasingly been developed to combine biological, physicochemical, hydromorphological, and governance dimensions into broader evaluations of freshwater system health (Esmaeili Ofogh et al., 2024; Reyes-Celis et al., 2025). One such approach is the pressure–condition–response (PCR) framework, which provides a coherent structure for integrating anthropogenic pressures, environmental conditions, and management responses into unified diagnostic assessments (Gayen & Datta, 2023). Within this perspective, the Tropical Water Health Index (TWHI) was designed specifically for tropical basins and combines indicators of land use, hydromorphology, water quality, biological integrity, and governance, enabling a more comprehensive assessment of water system health (Campos et al., 2024).

A key advantage of the TWHI lies in its operational flexibility; weighting and aggregation rules can be adapted to prioritize stream segments, diagnose critical bottlenecks, and guide interventions such as diffuse pollution control, riparian restoration, or enforcement of environmental compliance (Campos et al., 2024). However, for robustness and transferability, the TWHI requires ecoregional calibration, external validation under extreme conditions, sensitivity and uncertainty analyses, and iterative updates that integrate high-frequency data while maintaining comparability with historical records (Esmaeili Ofogh et al., 2024; Ruaro et al., 2024). In subtropical Brazil, urban water-supply basins represent mosaics of land use encompassing agroindustry, intensive agriculture, and expanding urbanization. Their juxtaposition generates diffuse, yet spatially heterogeneous pressures on water quality and ecosystem functioning (Bacca et al., 2023; Rezende et al., 2014).

These subtropical Brazilian basins are pivotal to water security, defined as the reliable provision of water in adequate quantity and quality, protection of aquatic ecosystems, and capacity to withstand climatic and anthropogenic shocks (Ballarin et al., 2023). Therefore, ecosystem-based management frameworks have been advocated to operationalize aquatic ecosystem governance by linking ecological indicators, water quality monitoring, and regulatory instruments within coherent, scalable decision processes (Koehler, 2023). The basin examined in this study is a critical drinking water source exhibiting pronounced longitudinal heterogeneity in land use and anthropogenic pressure. This diversity of conditions provides a natural experiment to assess how interacting stressors translate into physicochemical variability and biological responses and to derive management criteria consistent with integrated aquatic ecosystem management (Ballarin et al., 2023; Bohn et al., 2024; Koehler, 2023; Siqueira et al., 2023; Vieira & Ribeiro, 2022).

By integrating sensitive bioindicators, analytical tools capable of detecting nonlinearities and interactions, and a decision-oriented framework, this study aims to (i) elucidate how environmental gradients structure aquatic communities in a subtropical drinking-water basin, (ii) identify ecologically meaningful thresholds with direct management relevance, and (iii) evaluate the capacity of multicriteria syntheses to translate ecological evidence into actionable guidance for regional water security. The first premise of this study is that subtropical streams, which are essential to regional water supply, are simultaneously exposed to multiple environmental stressors whose interactions can generate nonlinear ecological responses (Huang et al., 2025; Su et al., 2025). Within this multidimensional context, water temperature is expected to function as a dominant environmental driver and a central axis around which community organization is structured, because it directly regulates organismal metabolism while simultaneously influencing oxygen availability, nutrient cycling, microbial activity, and other physicochemical processes that shape habitat suitability and community persistence (Han et al., 2025; Rezende et al., 2014; Sultana et al., 2020).

In contrast, conductivity and substrate heterogeneity are expected to influence community organization through distinct but complementary mechanisms. Conductivity reflects changes in ionic concentration and anthropogenic disturbance, potentially constraining sensitive taxa through osmotic and chemical stress, whereas substrate heterogeneity increases habitat complexity and niche availability, promoting biodiversity and ecological stability (Rezende et al., 2014). Accordingly, we hypothesized that (i) temperature is the primary driver structuring aquatic communities, due to its simultaneous influence on physiological and biogeochemical processes; (ii) biodiversity decreases under elevated conductivity because of increasing environmental stress; (iii) biodiversity increases with substrate heterogeneity as habitat complexity expands ecological niche availability; and (iv) taxon-specific ecological thresholds emerge along these gradients, reflecting differences in physiological tolerance and habitat specialization.

## Material and methods

### Study area

The research was carried out in the Lajeado São José (LSJ) micro-watershed, located in the Uruguay River basin and draining into the Prata River system. This catchment covers approximately 7744 hectares and is situated between 575 and 675 m in elevation within the municipalities of Chapecó and Cordilheira Alta in Santa Catarina, southern Brazil. Its geographical boundaries extend from 52°35′31″ to 52° 41′ 34″ W and from 26°58′40″ to 27° 07′ 00″ S. The region’s climate is classified as super-humid mesothermal, featuring high precipitation levels and elevated evapotranspiration rates typical of subtropical zones. The native vegetation is primarily composed of subtropical evergreen forests. The soil types are mainly Bruno-Roxo Álico Oxisols and Eutrophic Cambisols.

Land use in the LSJ micro-watershed presents a diverse landscape composed of forest remnants, agricultural plots (mainly corn and soybean), and grazing areas. Urban growth began in the 1970 s with the emergence of agro-industrial activities and expanded further in the 1980 s with the establishment of new residential zones. Currently, this watershed is a critical water source for the city of Chapecó, supplying treated water for consumption. Additionally, the remaining native vegetation contributes to biodiversity conservation and supports essential ecosystem functions, such as water regulation and habitat provision. The watershed was predominantly characterized by agricultural land use, which represented, on average, 60.19% of the surrounding landscape across sampling sites. Forest cover accounted for 18.17%, followed by urban areas (16.67%), silviculture plantations (3.22%), and exposed soil areas (0.57%).

**Fig. 1:**
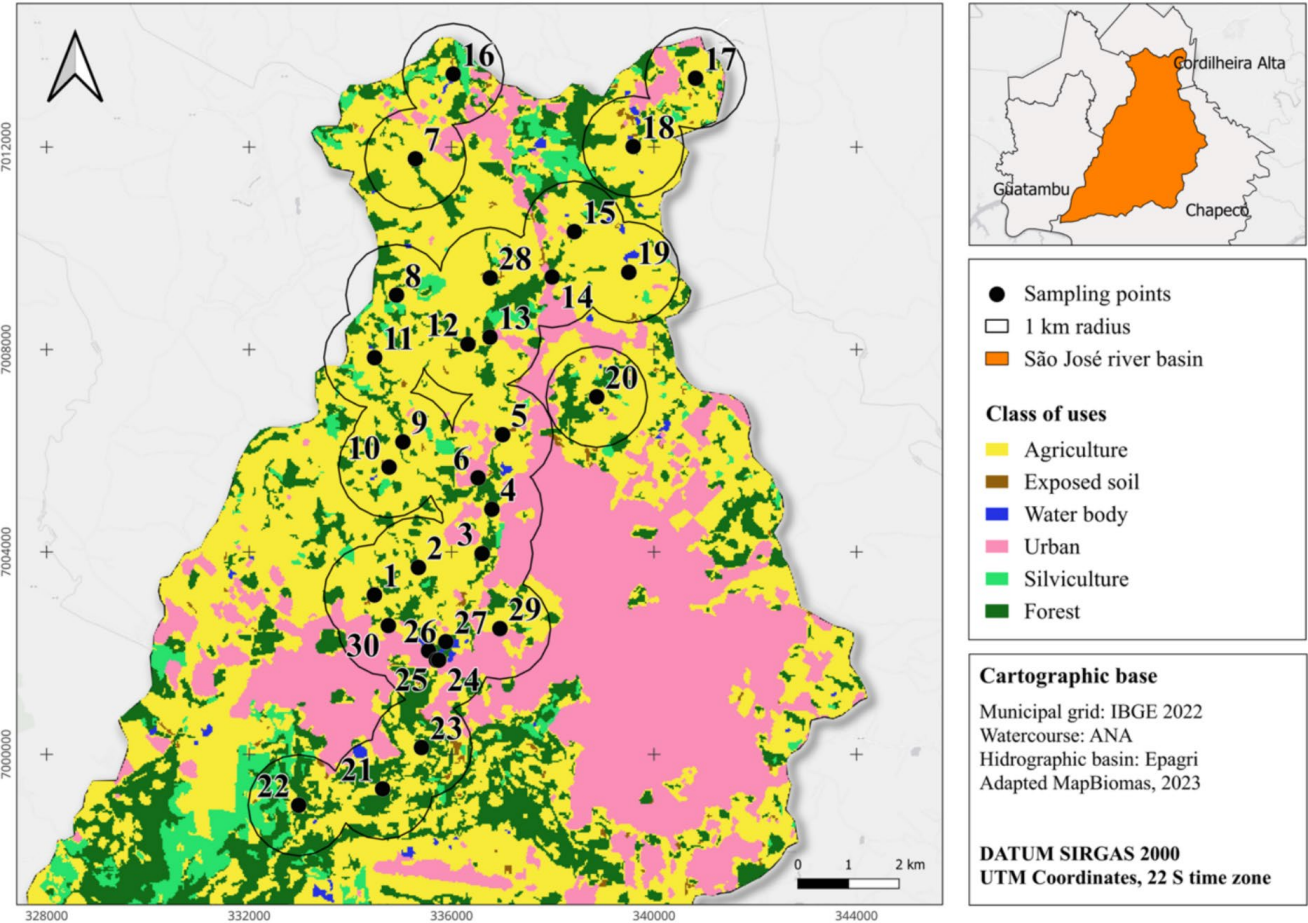
Spatial distribution of sampling points (red circles) in the São José River Basin (orange, upper right inset), covering different land-use and land-cover classes. The classes included agriculture (yellow), exposed soil (brown), water bodies (blue), urban areas (pink), silviculture (light green), and forest (dark green). Black circles indicate 1-km radius buffers around each sampling point. The location of the study area in Brazil is shown in the inset map. Cartographic base: municipal grid (IBGE, 2022), hydrography (ANA), and watershed boundaries (Epagri). Reference system: SIRGAS 2000, UTM coordinates, zone 22S

Sampling locations were selected to represent the full longitudinal profile of the Lajeado São José micro-watershed from its headwaters to downstream reaches beyond the urban boundary of Chapecó, Brazil. Thirty sites were distributed across the basin, considering logistical accessibility, habitat representativeness, and the presence of environmental gradients, including varying degrees of anthropogenic influence (Fig. 1; Table SM1).

### Procedures

The physicochemical parameters were measured in situ using a multiprobe meter (YSI Model 85). Measurements were consistently obtained in the center of the river flow at each sampling site to minimize the influence of marginal pools or low-flow depositional areas on physicochemical variability. The measured variables included water temperature (Temp), pH, oxidation–reduction potential (ORP), electrical conductivity (EC), turbidity (TRB), total dissolved solids (TDS), dissolved oxygen saturation percentage (DO), and total suspended solids (SPC). Surface water was also collected for the laboratory determination of nitrate (NO_3_⁻) and nitrite (NO_2_⁻) concentrations via UV–visible spectrophotometry, following established protocols (Table SM1).

Fluvial sediment texture was assessed using dry mechanical sieving, and the grains were classified into seven size fractions: stone (> 4.75 mm), pebble (> 2.30 mm), very coarse sand (> 1.40 mm), coarse sand (> 0.250 mm), medium sand (> 0.150 mm), fine sand (> 0.053 mm), and silt/clay (< 0.053 mm). The relative composition of each class was calculated based on the percentage of the total dry sample weight (Table SM2). Sediment grain size composition was assessed following the protocol of Suguio (1973), as modified by Rezende et al. (2014).

### Benthic macroinvertebrates and BMWP index

Benthic macroinvertebrates were sampled using a Surber sampler (1024-cm^2^ area and 0.250-mm mesh size). Three replicate samples were collected from each site such as one from each bank and one from the mid-channel in two campaigns (summer and winter), totaling 180 samples across all sites. Sampling was conducted across heterogeneous substrates to capture local habitat variability and improve the representativeness of benthic assemblages. The collected specimens were preserved in 70% ethanol and transported to the laboratory, where they were rinsed through a 0.50-mm mesh sieve and sorted under a stereomicroscope. Taxonomic identification at the family level followed standard references (Cummins, 1996; Hamada & Ferreira-Keppler, 2012; Hamada et al., 2014).

We used the Biological Monitoring Working Party index (BMWP) to assess water quality, as adapted by the Paraná Environmental Institute. This biotic index is based on taxonomic richness and the presence or absence of benthic macroinvertebrates in the samples. Organisms identified at the family level are assigned tolerance scores ranging from 1 to 10, reflecting their sensitivity to organic pollution. More sensitive families receive higher scores, whereas those tolerant of organic contaminants are assigned lower values.

### Phytoplankton

Subsurface phytoplankton samples were collected at all 30 sites during both the summer and winter campaigns. For qualitative assessment of community composition, horizontal tows were conducted using a 20-µm mesh plankton net, dragged just below the water surface for approximately four minutes. Samples were preserved in the field with 3% Lugol’s iodine solution and transported to the laboratory for taxonomic analysis. Phytoplankton were identified at the genus level using a standard optical microscope, with the aid of specialized literature (Bicudo & Menezes, 2006; Prescott et al., 1977, 1982) and recent taxonomic articles describing new genera.

### Predictor selection

To identify environmental drivers and characterize biological responses across gradients, we adopted a sequential modeling workflow integrating random forest (RF), boosted regression trees (BRT), and threshold indicator taxa analysis (TITAN). Each analytical component addresses a distinct ecological question and provides complementary information rather than redundant outputs. Random forest models (aided by boosted regression trees (BRT)) were first used as a screening procedure to determine the relative importance of environmental predictors influencing macroinvertebrate and phytoplankton taxa. Random forest is particularly suited for ecological datasets with nonlinear responses and complex interactions, while remaining robust to noise and nonnormal distributions (Breiman, 2001). Boosted regression trees were subsequently applied to quantify the shape of the relationships between key predictors and community metrics. Boosted regression tree models combine regression trees with boosting algorithms to capture nonlinear responses and high-order interactions, allowing the visualization of response curves through partial dependence plots (Elith et al., 2008). Finally, threshold indicator taxa analysis (TITAN) was used to detect taxon-specific change points along the most influential environmental gradients identified in the RF and BRT analyses. TITAN integrates change-point analysis with indicator species statistics, enabling the identification of ecological thresholds based on coherent shifts in species frequency and abundance (King et al., 2011).

This sequential framework therefore separates predictor identification (RF), response characterization (BRT), and threshold detection (TITAN), reducing redundancy while improving ecological interpretability. Before model fitting, spatial autocorrelation in model residuals was assessed using Moran’s *I* statistics based on geographic coordinates of sampling sites. Because the dataset included two seasonal sampling campaigns across a longitudinal gradient, sampling period was included as a blocking factor in exploratory analyses to ensure that major environmental patterns were not driven solely by seasonal differences.

### Environmental predictor selection

To identify the most influential environmental predictors shaping biological responses, we combined boosted regression trees (BRT) and random forest (RF) modeling approaches in R version 4.4.1 (R Core Team, 2025). For the BRT analysis, we used the gbm.step function from the gbm package. BRT models iteratively combine multiple regression trees using a gradient boosting algorithm, effectively capturing nonlinear relationships and high-order interactions (Friedman, 2001). The model parameters were standardized across the response variables as follows: tree complexity = 5, learning rate = 0.001, and a minimum of 1000 trees, as recommended by Elith et al. (2008). A bag fraction of 0.5 was used to introduce stochasticity by sampling 50% of the training data without replacement for each iteration. The model performance was evaluated using tenfold cross-validation, yielding the proportion of deviance explained (training performance), cross-validated correlation (testing performance), and relative influence of each predictor (summing to 100%). Partial dependence plots were generated to visualize the marginal effect of each predictor, illustrating linear, curvilinear, and threshold-type relationships.

For the RF analysis, we applied the ranger function (package ranger) to each taxon of the macroinvertebrate and phytoplankton datasets (1000 trees; mtry = √*p*), computing permutation-based variable importance following Breiman (2001). Model runs were parallelized to ensure reproducibility via the L’Ecuyer CMRG random seed method. Models with out-of-bag (OOB) errors exceeding one standard deviation of the corresponding response variables were excluded from further analysis. The global importance of each environmental predictor *k* was then estimated as the arithmetic mean of its permuted importance across m valid models (one per taxon): *VI*_*k*_ = 1/*m*_*j*=1_Σ^*m*^ *VI*_*kj*;_ where *VI*_*kj*_ denotes the permuted importance of predictor *k* in the model fitted to taxon *j*. This aggregation provides a community-level estimate of the predictor relevance while minimizing the influence of outliers. The predictive performance was assessed by computing the coefficient of determination (*R*^2^), observed–redicted correlation, residual mean squared error (MSE), and total deviance. These statistics were synthesized to estimate the global variance and mean training correlation with standard errors, providing robust measures of overall model performance and predictor influence across communities.

### Indicator taxa analysis along environmental gradients

To detect ecological thresholds along environmental gradients, we applied threshold indicator taxa analysis (TITAN) using the titan function from the “TITAN2” package in R (Baker & King, 2013). Separate models were run for macroinvertebrate communities, BMWP scores, and phytoplankton assemblages using the most influential environmental variables identified through BRT and random forest analysis. TITAN integrates principles from indicator species analysis (Dufrêne & Legendre, 1997) and change point analysis (King & Richardson, 2003) to detect shifts in species distributions based on changes in their frequency and abundance along continuous environmental gradients (King et al., 2011). This method identifies both negative (*z*-, decliners) and positive (*z* +, increasers) responders, corresponding to taxa that exhibit significant changes near the low and high ends of each gradient. Only taxa recorded in at least three sampling plots were considered. Thresholds were considered statistically robust when indicator values were significant (IndVal *p* ≤ 0.05) and met the minimum criteria for purity and reliability (≥ 0.70).

### A customization index by cost and ecological efficiency indicators

To complement bioindicator and multivariate analyses, we applied an adapted version of the Tropical Water Health Index (TWHI) developed within the pressure–condition–response (PCR) framework. The TWHI was designed for full customization to regional contexts, enabling its application in data-limited and heterogeneous tropical basins worldwide. The index integrates multiple environmental dimensions by combining pressure, efficiency, and response indicators derived from the most influential variables identified in the BRT, random forest, and TITAN analyses. All indicators were standardized to a [0,1] scale using fuzzy membership functions (harmony scores) and aggregated into weighted PCR layers, following the procedures described by Campos et al. (2021, 2024).

Standardization to the [0,1] range was performed using five-node trapezoidal membership functions, corresponding to qualitative levels of ecosystem condition (0.0–0.2 = Critical, 0.2–0.4 = Poor, 0.4–0.6 = Fair, 0.6–0.8 = Good, and 0.8–1.0 = Very good). For efficiency indicators (higher values = better condition), harmony increased linearly from 0 (*Critical*) to 1 (*Fair*) and symmetrically declined toward 0 (*Very good*), producing a bell-shaped trapezoid centered at *c*. For cost indicators (higher = worse condition), a mirrored function was used, where low values yielded high harmony, and high values progressively reduced harmony. For indicators with an internal optimum (*x_best ≈ c*), a U-shaped function was applied, where harmony decreased with the absolute distance from the optimum, reflecting variables with maximum ecological performance at intermediate levels. Legal compliance indicators (SCU) were directly converted to discrete harmony scores via linear mapping, assigning values of 1.0, 0.8, 0.6, 0.4, and 0.2 to the categories *Critical* through *Very good*, respectively.

This fuzzy logic-based framework (i) integrates multidimensional ecological and regulatory information into a unified, scalable metric; (ii) balances efficiency and pressure indicators while maintaining interpretability; and (iii) ensures methodological flexibility for adaptation to basin-specific environmental and legal contexts. Such a design makes the TWHI particularly useful for diagnosing ecosystem conditions in anthropogenically impacted tropical basins with relatively homogeneous land-use patterns.

## Results

### Aquatic communities

A total of 6398 aquatic macroinvertebrates were recorded across the 30 sampling stretches of streams, encompassing 49 families (Table SM3). The families with the highest total abundance were Chironomidae (2634 individuals), Baetidae (758), Hydropsychidae (676), and Hirudinidae (426), which together accounted for approximately 70% of all individuals sampled. In terms of site richness, the number of families detected per site ranged from 9 to 25, with stretch 26 showing the highest richness and stretch 3 and 25 the lowest, respectively. The most frequent families were Chironomidae (present at 28 stretches), Baetidade (28 stretches), Hydropsychidae (28 stretches), and Hurudinidae (26 sites). The BMWP (Biological Monitoring Working Party; Table SM3) index values, used as a proxy for water quality, ranged from 17 (stretch 3) to 98 (stretch 26), with a mean value of approximately 50. Sites with higher BMWP scores were characterized by the presence of sensitive taxa such as Baetidae, Calopterygidae, and Leptophlebiidae, whose occurrence directly contributed to the elevated biotic integrity values recorded at those locations. The families Ceratopogonidae, Coenagrionidae, and Oligochaeta also showed moderate frequencies across sites, suggesting tolerance to a range of environmental conditions. In contrast, taxa such as Tabanidae, Scirtidae, and Tipulidae were recorded at only one or two stretches.

A total of 25 phytoplankton genera were recorded across the 30 sampling sites (Table SM4). The most widespread taxa in terms of occurrence were Closterium (present at 23 sites), *Surirella* (21 sites), and *Desmodesmus* (19 sites), which collectively represented the most consistently distributed genera across the landscape. These three taxa occurred at over 70% of all sampling points, indicating a high degree of environmental tolerance and ecological generalism. Conversely, several genera displayed restricted distributions, occurring at fewer than four sites. These included *Micrasterias*, *Tetraedriella*, *Tetraedron*, *Tetrastrum*, *Komvophoron*, *Stauridium* and *Spirogyra*. Notably, *Staurastrum*, *Tropidoscyphus, Hariotina, Cosmarium, Crucigenia, Treubaria *and *Westella* were present at only four or five sites. On average, each site harbored approximately seven to ten genera, with higher richness generally observed in upstream and intermediate stretches characterized by greater habitat heterogeneity and lower local anthropogenic influence, such as P10, P12, and P28. In contrast, lower richness tended to occur at more environmentally simplified or impacted stretches, including P1, P3, P17 and P20. We found the presence of multiple desmid genera, such as *Euastrum*, *Cosmarium*, and *Staurastrum*, at selected sites. The occurrence of cyanobacteria, such as Spirulina, was also recorded, although at relatively low frequency (six sites).

### Relative contributions of environmental variables on community level

**Fig. 2:**
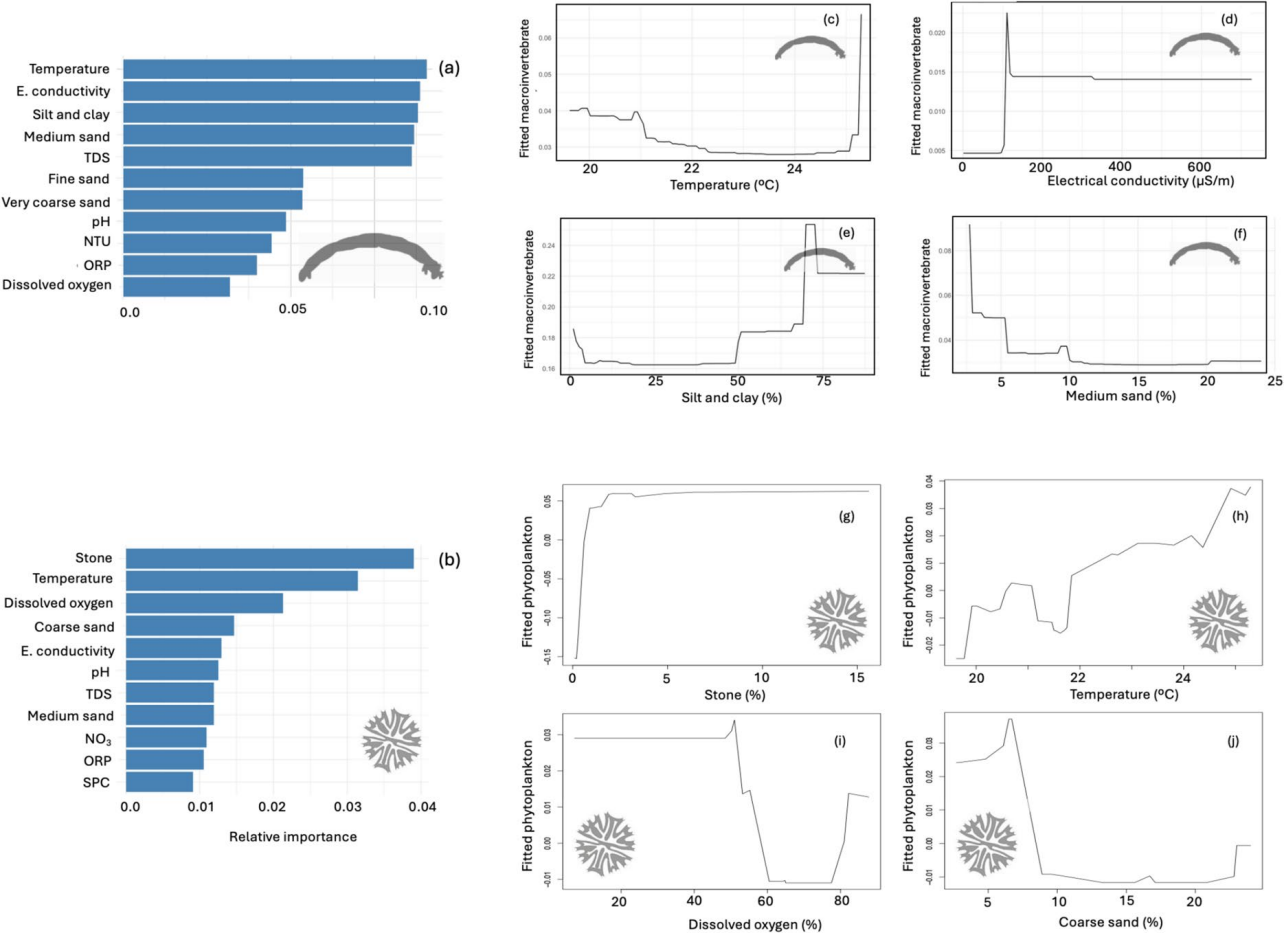
Random forest models from average permuted importance over m valid models of the macroinvertebrate (**a**) and phytoplankton (**b**) communities. In addition, the fitted functions for the best performance models of the macroinvertebrate (**c**–**f**) and phytoplankton (**g**–**j**) communities are shown. The plots show only the four most relevant variables for the explained deviance in the metric. Rug plots show the distribution of data in deciles of the variable on the *X*-axis

Random forest models based on the average permuted importance across *m* valid models for each macroinvertebrate taxon identified water temperature as the most influential environmental predictor, followed by electrical conductivity, silt and clay fractions in the streambed, intermediate-sized substrate particles, and total dissolved solids (TDS) (Fig. 2a; each of approximately 10% importance; Table SM5). Community-level frequency and occurrence declined along the water temperature gradient, with a slight resurgence beyond 25 °C (Fig. 2c). The electrical conductivity exhibited a unimodal pattern, with a peak near 100 µS·m^−1^, followed by a plateau (Fig. 2d). A peak in macroinvertebrate frequency and occurrence was observed at sites where silt and clay comprised approximately 75% of the substrate (Fig. 2e). In contrast, medium sand fractions had an adverse effect at > 5% coverage (Fig. 2f).

Random forest models based on the average permuted importance across *m* valid models for each phytoplankton taxon revealed substrate cover by stones (± 4% importance) as the primary predictor, followed by water temperature (± 3% importance), dissolved oxygen (± 2% importance), and coarse substrate area (± 1.5% importance; Fig. 2b; Table SM5). Phytoplankton frequency and occurrence increased with the proportion of stones in the substrate and plateaued after reaching 3% stone cover (Fig. 2g). Community-level frequency and occurrence also increased steadily along the temperature gradient, with no upper threshold observed within the measured range (Fig. 2h). A notable decline in phytoplankton levels was detected between 60 and 75% dissolved oxygen saturation (Fig. 2i). In addition, coarse sand fractions harmed community levels when they comprised approximately 10% of the substrate (Fig. 2j).

**Fig. 3:**
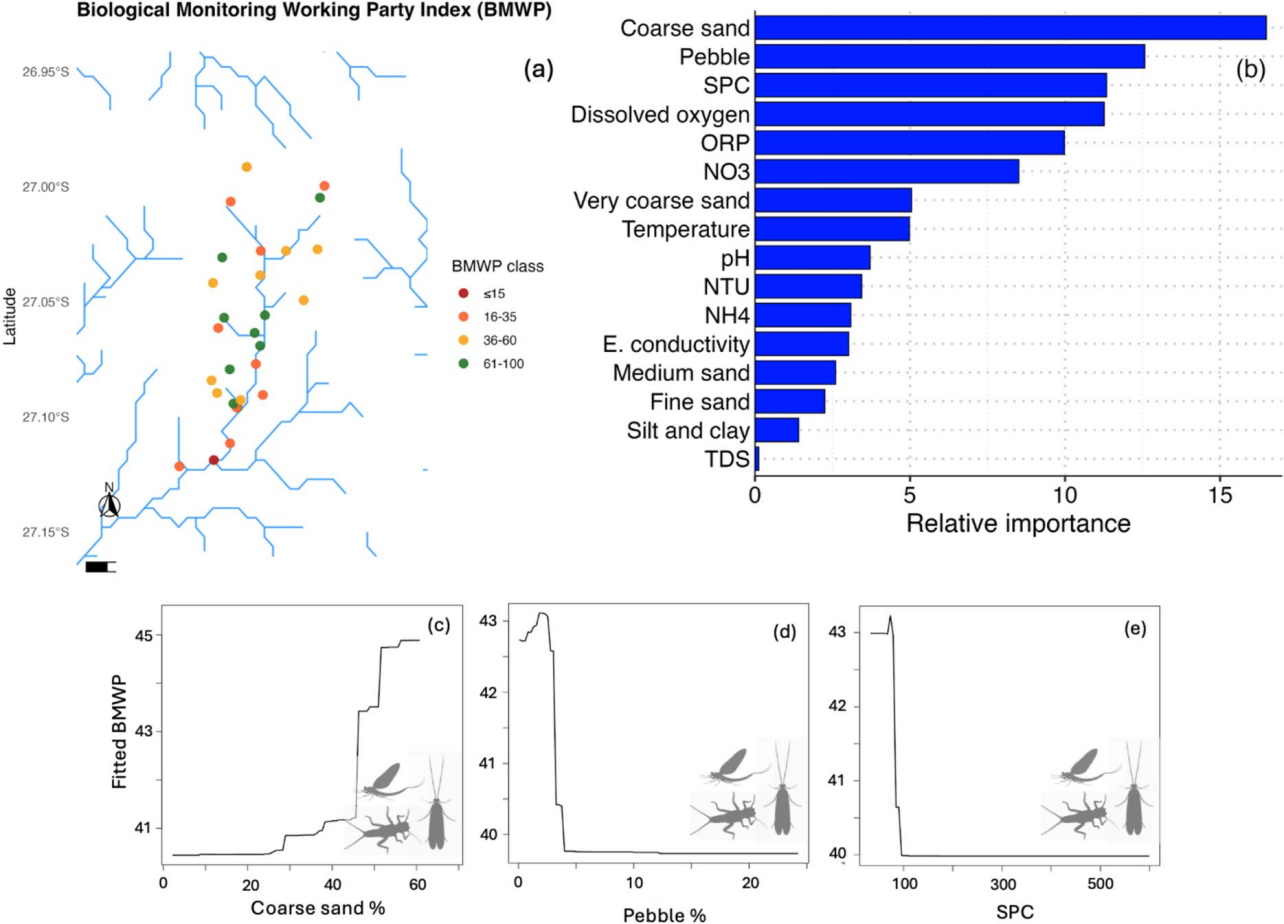
Geographic distribution of the BMWP index in the study sampling areas (**a**). Boosted regression tree (BRT) fitted functions for the best-performing models of the BMWP index (**b**). Plots show only the three most relevant variables for explained deviance in the metric (**c**–**e**). Rug plots show the distribution of data, in deciles, of the variable on the *X*-axis (**c**–**e**)

Boosted regression tree (BRT) of the BMWP index (Table SM5; Fig. 3a) revealed that coarse sand (~ 15% importance), cobbles (~ 22%), suspended particulate concentration (SPC), and dissolved oxygen (each ~ 11%) were the most influential environmental predictors (Fig. 3b). BMWP scores increased markedly when coarse sand in the streambed exceeded approximately 45% (Fig. 3c). In contrast, the index declined beyond approximately 5% pebble content (Fig. 3d) and at suspended particulate concentrations of approximately 100 units (Fig. 3e).

Stream discharge exhibited substantial spatial variability across the basin during both sampling campaigns, reflecting differences in channel size, hydrological connectivity, and local geomorphological conditions. Discharge values ranged from 0.10 to 38.72 m^3^·s⁻^1^, although most stretches exhibited relatively low discharge conditions (< 10 m^3^·s⁻^1^), consistent with low- to medium-order subtropical streams under baseflow conditions. Higher discharge values were observed at sites such as P3, P14, P21, P23, P24, and P28 during at least one sampling campaign. Importantly, no precipitation events occurred during the two weeks preceding either sampling campaign, suggesting that the measured hydrological conditions likely reflected relatively stable baseflow conditions rather than short-term runoff responses.

### Relationships between taxa and key environmental predictors

**Fig. 4:**
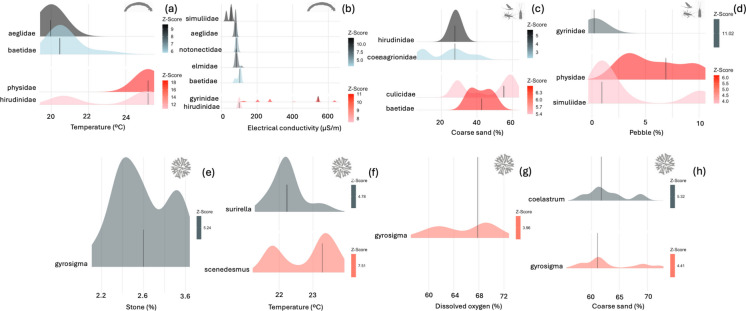
Results of the TITAN of the macroinvertebrate community by temperature (**a**), water electrical conductivity (**b**), percentage of coarse sand in sediment stream (**c**), and percentage of pebbles in sediment stream (**d**) and phytoplankton community by percentage of stones in sediment stream (**e**), water temperature (**f**), water dissolved-oxygen (**g**), and percentage of coarse sand in sediment stream (**h**) along watershed basin. Robust sensitive taxa (*Z*-) and tolerant taxa (*Z* +) are represented by blue and red scales, respectively. The blue/red color intensity is proportional to the magnitude of the response. Black vertical bars = change point of each functional trait. The area below the curve represents the 5th and 95th percentiles among 500 bootstrap replicates

Threshold indicator taxa analysis (TITAN) of the macroinvertebrate community (Table SM6) revealed that water temperature was the primary driver of multiple taxa (Fig. 4a). Aeglidae and Baetidae exhibited strong negative thresholds near 20–21 °C, indicating thermal sensitivity. In contrast, Physidae and Hirudinidae responded positively to higher temperatures (23–24 °C), suggesting a tolerance for or preference toward warmer waters. Conductivity was also a key gradient (Fig. 4b). Simuliidae, Aeglidae, Notonectidae, Elmidae, and Baetidae all showed negative associations with rising conductivity, favoring low-ionic strength environments (< 200 µS·m^−1^). Conversely, Gyrinidae and Hirudinidae were positively associated with higher conductivity (500 µS·m^−1^). Substrate composition also influenced the taxon responses. Hirudinidae and Ceratopogonidae exhibited declines in areas with high coarse sand content, implying a preference for finer or more stable sediments (Fig. 4c). Baetidae and Culicidae showed increased occurrence in substrates with > 40% coarse sand, indicating adaptation to more mobile or physically dynamic conditions. Lastly, Gyrinidae demonstrated a sharp negative threshold near 0% cobble cover, whereas Physidae and Simuliidae responded positively to modest cobble presence (4–7%; Fig. 4d), suggesting either preference or tolerance for more structured benthic habitats.

TITAN analysis of the phytoplankton assemblage (Table SM7) also revealed taxon-specific thresholds in response to physical and chemical gradients. *Gyrosigma* showed a strong positive response to streambed stone cover, with a significant threshold at approximately 2.6%, indicating a preference for moderately structured habitat (Fig. 4e). *Surirella* exhibited a negative threshold near 22.5 °C, favoring cooler waters, whereas *Scenedesmus* responded positively around 23 °C, consistent with a preference for warmer, potentially nutrient-rich conditions (Fig. 4f). *Gyrosigma* also showed a positive threshold near 68% dissolved oxygen saturation (Fig. 4g), suggesting affinity for well-oxygenated environments potentially linked to high photosynthetic activity or low organic load. With respect to substrate texture, *Coelastrum* exhibited a negative response to increasing coarse sand. In contrast, *Gyrosigma* again showed a strong positive threshold at approximately 65–70% coarse sand (Fig. 4h), reinforcing its association with heterogeneous and structured sediment environments.

### Tropical Water Health Index

**Fig. 5:**
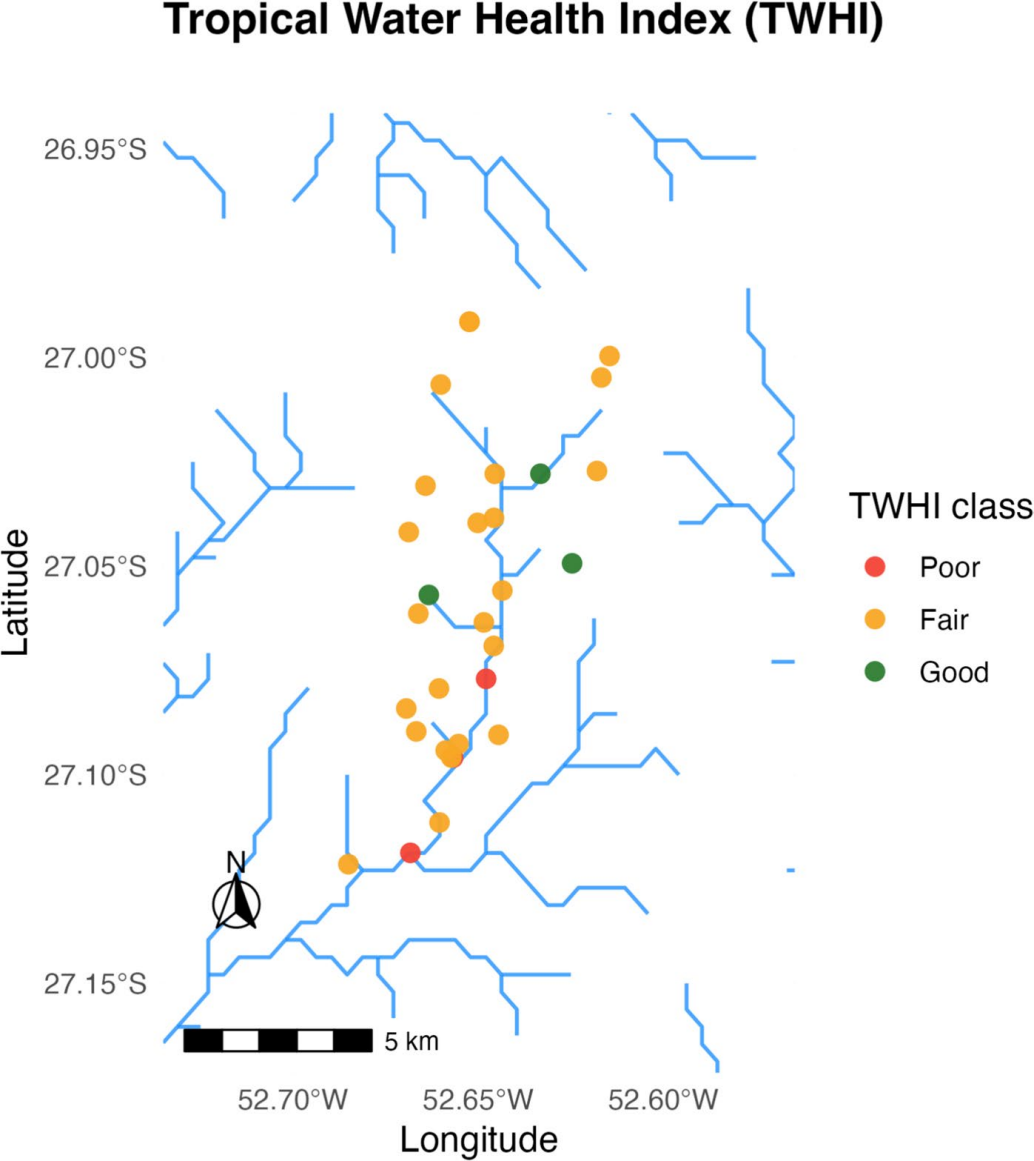
Spatial distribution of the Tropical Water Health Index (TWHI) across 30 georeferenced sampling sites based on cost and efficiency indicators

The adapted Tropical Water Health Index (TWHI; Fig. 5) for the 30 sampling sites revealed a predominance of intermediate classifications (Table SM8). Most sites (*n* = 23; 76.7%) were classified as *Fair*, with index values ranging from 0.44 to 0.59. The second most frequent category was *Poor* (*n* = 3; 10.0%), representing the lowest quality scores (0.37–0.40), followed by *Good* (*n* = 4; 13.3%), which concentrated the highest scores (0.60–0.63). No sites were classified as *Critical* or *Very good*, indicating the absence of extreme degradation or exceptional quality conditions (Fig. 5; Table SM8 and automation in Script SM9).

## Discussion

### Overview

This study presents an integrative framework to infer how interacting environmental gradients, including temperature, conductivity, and habitat structure, jointly shape aquatic community organization across subtropical fluvial networks. By combining ecological theory with operational diagnostic approaches, the framework identifies nonlinear biological responses with direct implications for freshwater conservation and adaptive management. The results indicate that ecological vulnerability does not emerge from isolated environmental gradients, but rather from the interplay among thermal conditions, oxygen dynamics, and habitat simplification operating across multiple spatial and functional scales (Nuven et al., 2022; Rezende et al., 2014). Collectively, these findings may provide an evidence-based foundation for strengthening water governance and water security under ongoing environmental change, while enabling the early detection of ecological instability in thermally sensitive freshwater ecosystems.

Water temperature emerged as the dominant environmental predictor associated with aquatic community structure throughout the investigated subtropical basin, exerting greater explanatory influence than electrical conductivity and streambed morphology under the sampled environmental conditions. Increases in measured water temperature coincided with pronounced compositional reorganization, with assemblages dominated by the families Baetidae and Elmidae occurring more frequently in cooler and less environmentally altered sites, whereas tolerant and generalist taxa, including those from Physidae family, were more frequently associated with warmer stretches subjected to greater environmental disturbance. These patterns are consistent with the increasingly widespread global signal of biodiversity restructuring and ecological erosion under intensifying thermal and anthropogenic pressures (Bonacina et al., 2023; Johnson et al., 2024; Lee et al., 2025a, 2025b).

Within primary producer assemblages, higher measured temperatures coincided with greater occurrence of opportunistic chlorophytes, corroborating previous studies reporting shifts in phytoplankton composition under warmer and nutrient-enriched conditions (Bao et al., 2022; Bowes et al., 2024; Hattich et al., 2024; Juffermans et al., 2025; Rossi et al., 2023; Songserm et al., 2024). Together, these findings reinforce the concept of interacting environmental gradients, whereby thermal conditions interact with chemical and geomorphological variables to structure aquatic biota across trophic and organizational levels (Campos et al., 2021, 2022). Even relatively modest thermal variation may therefore coincide with broad ecological reorganization, reshaping community composition, trophic dynamics, and ecosystem functioning (Martins et al., 2017; Navarro et al., 2013). Anticipating the ecological consequences of ongoing regional warming thus requires integrative approaches capable of resolving the feedback among temperature, water chemistry, and habitat structure within subtropical freshwater networks.

The combined application of random forest (RF), boosted regression trees (BRT), and threshold indicator taxa analysis (TITAN) generated complementary rather than redundant ecological insights. Machine learning approaches identified the dominant environmental filters structuring aquatic communities and characterized nonlinear response trajectories at the assemblage level, whereas TITAN detected taxon-specific thresholds along the same environmental gradients. This multistep analytical framework enabled the transition from predictor identification to the ecological interpretation of threshold-associated responses, representing a critical step toward translating complex ecological signals into management-relevant indicators for freshwater monitoring and conservation planning.

### Nonlinear ionic and substrate-mediated responses

Electrical conductivity and substrate composition jointly modulated the relationships associated with temperature, producing complex and frequently nonlinear diversity responses across the subtropical streams. Maximum macroinvertebrate richness was observed at intermediate conductivity values, consistent with the “ionic optimum” hypothesis, whereby low ionic strength may constrain biological activity, whereas high conductivity is frequently associated with diffuse nutrient enrichment and anthropogenic disturbance (Campos et al., 2022; Feng et al., 2025). Because conductivity represents an aggregate measure of dissolved ions rather than ionic composition itself, the observed relationships should be interpreted as associations with overall ionic conditions rather than responses to specific ions or solutes. Sediments with moderate proportions of silt and clay supported greater organismal abundance by providing relatively stable depositional areas, fine-scale refugia, and organic detritus retention (Mathers et al., 2024; McKenzie et al., 2024), whereas substrates dominated by medium sand exhibited reduced richness, likely reflecting greater physical instability and lower organic matter retention capacity (Chi et al., 2024; Lee et al., 2025a, 2025b; Vallefuoco et al., 2024).

Intermediate cobble coverage enhanced microhabitat heterogeneity and hydraulic complexity, resulting in elevated Biological Monitoring Working Party (BMWP) scores, while excessive coarse substrate coincided with reductions in both structural stability and ecological performance (Mathers et al., 2024). In contrast, high concentrations of suspended solids (TSS/SPC) were associated with reduced community integrity, potentially through the obstruction of interstitial spaces and impaired gas and nutrient exchange (Mendoza et al., 2025; Williams & Efta, 2024). Dissolved oxygen (DO) emerged as one of the most important physicochemical predictors associated with biological integrity across the basin. Lower DO values coincided with reductions in BMWP scores, declines in Ephemeroptera–Plecoptera–Trichoptera (EPT) richness, and greater occurrence of tolerant taxa such as organisms from Chironomidae and Hirudinidae families (Liang et al., 2024; Mendoza et al., 2025). Although the present study did not directly evaluate physiological performance or metabolic responses, these associations are consistent with previous ecological studies showing that oxygen availability is closely linked to habitat suitability for many aquatic taxa, particularly EPT taxa commonly associated with well-oxygenated streams (Bonacina et al., 2023; Liang et al., 2024). The phytoplankton responses observed within the 60–75% oxygen saturation window further suggest that relatively small changes in oxygen dynamics may trigger ecological reorganization in subtropical streams. Collectively, these results highlight the interactive roles of conductivity, substrate structure, and oxygen availability in regulating stream biodiversity (Fanelli et al., 2024; Liang et al., 2024; McKenzie et al., 2024). These results underscore the necessity of region-specific calibration and careful mechanistic interpretation of interacting environmental gradients in tropical and subtropical freshwater assessments (Hora Revilla et al., 2025; Mureithi et al., 2025).

### Threshold responses

Under escalating climatic variability, threshold analyses derived from TITAN revealed distinct taxon-specific responses along both thermal and conductivity gradients. Along the temperature axis, Aeglidae and Baetidae exhibited reduced occurrence above approximately 20–21 °C, whereas Physidae and Hirudinidae became more frequent at sites with measured temperatures near 23–24 °C (Bonacina et al., 2023; Cochran et al., 2023; Phillips et al., 2020). Collectively, these shifts suggest a progressive replacement of assemblages associated with cooler and more oxygenated conditions by more tolerant and generalist communities under warmer environmental conditions. However, because physicochemical variables were measured during discrete sampling campaigns, these patterns should be interpreted as ecological associations under the sampled conditions rather than direct evidence of physiological thermal constraints. Among phytoplankton, *Surirella* and *Scenedesmus* genera displayed slightly different response peaks (~ 22.5 °C vs. ~ 23 °C), suggesting distinct environmental associations between diatoms and chlorophytes (Hattich et al., 2024; Rossi et al., 2023).

Along the conductivity gradient, the reduced occurrence of Simuliidae, Aeglidae, and Notonectidae at higher conductivity values suggests associations with less mineralized or less environmentally altered conditions (Fanelli et al., 2024; Kaczmarek et al., 2024), while Gyrinidae and Hirudinidae remained frequent across a broader conductivity range. Substrate thresholds mirrored these gradients: Baetidae and Culicidae were more frequently associated with sandy reaches, whereas Hirudinidae and Ceratopogonidae declined under such conditions (Mathers et al., 2024; McKenzie et al., 2024). Intermediate cobble coverage enhanced niche diversification and surface flow opportunities (Rezende et al., 2014), coinciding with greater occurrence of Physidae and Simuliidae, which are commonly associated with periphyton-rich habitats and flowing water environments (Chi et al., 2024; Vallefuoco et al., 2024).

Among algal taxa, *Gyrosigma* responded positively to cobble substrates and moderate dissolved-oxygen levels (~ 68% saturation) but declined with coarse sand, whereas *Coelastrum* exhibited the inverse response, indicating distinct environmental associations between substrate retention and light-nutrient availability (Gao et al., 2024; Serôdio et al., 2023). Collectively, these taxon-specific thresholds illustrate how fine-scale physicochemical transitions may coincide with community reorganization under interacting environmental gradients (Davutoglu et al., 2024; Toskey et al., 2024), highlighting shifts in trophic structure and ecosystem functioning in subtropical streams (Gao et al., 2024; Serôdio et al., 2023).

### Implications for ecosystem management and water security

Two nonlinear relationships emerge with potential direct implications for basin-scale management strategies. First, the unimodal conductivity-diversity response underscores the need to define ecological “optima” rather than static thresholds, as both low and elevated conductivity conditions may coincide with reduced biodiversity patterns (Campos et al., 2022; Feng et al., 2025). Second, a dissolved oxygen (DO) window, approximately 60 to 75% saturation, coincided with an abrupt decline in phytoplankton abundance, potentially linked to diel oscillations masked by daily mean values in shallow, thermally dynamic streams (Jarvis et al., 2023; Tromboni et al., 2022; Zhu et al., 2024). These findings may reinforce the necessity of high-frequency monitoring to capture transient limit events and improve ecological interpretation in thermally dynamic freshwater systems.

The adapted Tropical Water Health Index (aTWHI) displayed sensitivity in detecting moderate impairment, although its resolution decreased at the extremes of the gradient, as reflected in the absence of *Very good* or *Critical* classes. This compression effect likely stems from (i) ceiling and floor constraints in key metrics (e.g., EPT and DO) (); Campos et al., 2022; King et al., 2011(ii) redundancy among correlated parameters (Schoolmaster et al., 2012); and (iii) insufficient temporal replication to capture ephemeral disturbances (Elfferich et al., 2024; Zhu et al., 2024). Nonetheless, by integrating physical, chemical, and biological components, the TWHI outperforms unidimensional indices, retaining stability under intermediate stress when calibrated regionally (Campos et al., 2022, 2024).

From a governance perspective, the aTWHI provides a pragmatic foundation for regulatory modernization in Brazil by embedding more biologically responsive criteria, particularly for extreme impairment or high-quality classes. Future refinements should incorporate high-sensitivity modules encompassing (i) functional indicators, such as trait diversity and tolerance ratios; (ii) high-frequency data streams capturing hypoxia, turbidity, and thermal peaks; and (iii) nonlinear scoring functions anchored in empirically derived ecological thresholds rather than percentile-based scaling. Such innovations would strengthen the index as a decision support tool for site classification, restoration planning, and hotspot detection within adaptive management frameworks to increase local water security.

### Valence and complementarity among ecological indices

The BMWP and TWHI indices reveal distinct yet complementary spatial signals, reflecting the contrasting philosophies behind a taxon-based index and an integrative, Pressure–Condition–Response framework. BMWP scores effectively captured localized shifts in macroinvertebrate sensitivity but tended to accentuate abrupt transitions near heavily impacted reaches (Hora Revilla et al., 2025), offering limited discrimination across intermediate conditions where multiple environmental gradients interact. This led, for example, to isolated *Good* classifications embedded within broader low-quality gradients (from “acceptable” to “doubtful” range).

In contrast, the TWHI generated a more continuous and spatially coherent pattern, mirroring the combined influence of land-use pressures, physicochemical conditions, and biological responses (Campos et al., 2021, 2022). Although less responsive to fine taxonomic turnover, its integrative structure captured broader environmental limitations, such as substrate simplification, thermal variability, and oxygen dynamics, that the BMWP alone may underrepresent. Accordingly, whereas the BMWP remains a robust tool for detecting changes in macroinvertebrate sensitivity, its single-dimension design offers a narrower view of how concurrent environmental gradients shape stream condition (Campos et al., 2022; Feng et al., 2025). The TWHI does not replace BMWP; rather, it complements it by providing a more holistic picture of ecosystem constraints, enhancing interpretation in systems where environmental drivers interact synergistically or subtly across multiple dimensions, helping with water security.

### Study limitations

Some limitations should be considered when interpreting the ecological relationships identified in this study. Physicochemical variables were measured during discrete sampling campaigns rather than through continuous high-frequency monitoring, which limits the ability to infer temporal variability and short-term environmental fluctuations, particularly for parameters such as water temperature and dissolved oxygen. Consequently, the observed relationships should be interpreted as ecological associations under the sampled conditions rather than direct mechanistic evidence of physiological constraints or causal responses. In addition, macroinvertebrates were identified at the family level, preventing species-specific inference regarding thermal tolerance, ecological specialization, or the occurrence of exotic taxa. Future studies integrating continuous environmental monitoring, species-level identification, and experimental approaches would help refine the mechanistic interpretation of ecological thresholds in subtropical freshwater systems.

## Conclusion

Water temperature emerged as the dominant environmental axis associated with aquatic community composition across the subtropical basin, exerting a stronger influence than conductivity and channel morphology under the sampled environmental conditions. Threshold analyses identified nonlinear ecological responses consistent with an intermediate conductivity optimum and a dissolved oxygen window between 60 and 75% saturation, both delineated by TITAN as important ecological transition zones associated with thermal, conductivity, and substrate gradients. Therefore, ecological vulnerability in these systems appears to emerge not from isolated environmental variables, but from the interplay among warming, oxygen dynamics, and habitat simplification, processes that collectively influence resilience, biotic integrity, and regional water security.

From a methodological perspective, the Tropical Water Health Index (TWHI) effectively detected moderate impairment but exhibited diminished sensitivity at the extremes of the gradient, reflecting the compression typical of multimetric indices based on linear scoring. Advancing this framework will require the integration of event-based and trait-based metrics, adoption of nonlinear, threshold-anchored calibration, and external validation emphasizing rare or transitional ecological states. When refined, the TWHI can evolve from a classification tool into a hybrid surveillance system capable of identifying ecological reference conditions and providing early warnings of functional decline. Collectively, these findings chart an evidence-based pathway toward adaptive water governance. By operationalizing nonlinear ecological insights into decision-making frameworks, the TWHI can support risk anticipation and intervention prioritization in thermally sensitive basins. The alignment of ecological diagnostics with institutional modernization offers a scalable, policy-relevant foundation for sustaining freshwater ecosystem integrity under accelerating climatic and anthropogenic pressures.

## Supplementary Information

Below is the link to the electronic supplementary material.

ESM 1 Supplementary Material 1 (DOCX 121 KB)

## Data Availability

The datasets generated and analyzed during the current study are available from the corresponding author upon reasonable request. The processed datasets and R scripts used for statistical analyses are available in script supplementary material 9.
