# Supplementary material for: From indicators to governance: a pressure–condition–response framework reveals nonlinear ecological responses to multiple stressors in an agricultural-urban basin
Source: Environ Monit Assess. 2026 Jul 2;198(7):799. doi: 10.1007/s10661-026-15608-3 (PMC13323321; doi:10.1007/s10661-026-15608-3)
Supplement: Supplementary file 1 — Supplementary Material 1 (DOCX 121 KB) [file 10661_2026_15608_MOESM1_ESM.docx]

**Table SM1.** Average values of water physicochemical parameters evaluated along the watershed basin. Temp.: water temperature; pH, ORP: Oxidation-Reduction Potential; EC: electrical conductivity; TRB: turbidity; TDS: total dissolved solids; DO: dissolved oxygen saturation percentage; SPC: total suspended solids; NO_2_⁻: nitrite and NO_3_⁻: nitrate.

| Site | Lat | Lon | Temp. (℃) | pH | ORP (mV) | EC (µS/cm) | TRB (NTU) | TDS (mg/L) | DO (%) | SPC (mg/L) | NO_2_⁻ (mg/L) | NO_3_⁻ (mg/L) |
| --- | --- | --- | --- | --- | --- | --- | --- | --- | --- | --- | --- | --- |
| 1 | -27.084116 | -52.669447 | 20.46 | 5.61 | 321 | 62 | 0.9 | 40 | 71.4 | 52.9 | 1.92 | 0.07 |
| 2 | -27.079319 | -52.660619 | 21.62 | 5.33 | 361 | 54 | 41.2 | 35 | 63.8 | 46.2 | 0.87 | 0.12 |
| 3 | -27.077 | -52.647889 | 21.19 | 5.96 | 249 | 93 | 162.0 | 61 | 48.5 | 84.5 | 2.63 | 0.34 |
| 4 | -27.069111 | -52.645833 | 21.07 | 6.26 | 297 | 98 | 7.0 | 63 | 68.8 | 82.7 | 2.64 | 0.23 |
| 5 | -27.055833 | -52.6435 | 21.75 | 7.10 | 228 | 103 | 3.5 | 67 | 68.8 | 88.5 | 3.93 | 0.06 |
| 6 | -27.063472 | -52.6485 | 19.62 | 6.45 | 200 | 76 | 102.0 | 49 | 70.0 | 64.4 | 1.61 | 0.09 |
| 7 | -27.006444 | -52.660139 | 19.92 | 6.29 | 192 | 80 | 2.2 | 52 | 87.7 | 67.3 | 1.58 | 0.11 |
| 8 | -27.030694 | -52.66425 | 21.84 | 7.44 | 284 | 72 | 54.1 | 47 | 60.5 | 64.9 | 0.38 | 0.04 |
| 9 | -27.056889 | -52.663361 | 20.29 | 6.37 | 252 | 102 | 8.8 | 66 | 64.8 | 82.7 | 3.45 | 0.09 |
| 10 | -27.061361 | -52.666194 | 22.62 | 6.27 | 269 | 95 | 159.0 | 61 | 38.2 | 97.3 | 2.38 | 0.19 |
| 11 | -27.041806 | -52.668778 | 24.37 | 7.11 | 280 | 102 | 5.3 | 66 | 68.9 | 86.5 | 0.98 | 0.08 |
| 12 | -27.039611 | -52.650167 | 21.06 | 6.72 | 266 | 111 | 20.6 | 72 | 64.2 | 93.1 | 3.29 | 0.37 |
| 13 | -27.038417 | -52.64575 | 19.68 | 7.03 | 293 | 79 | 2.2 | 51 | 81.0 | 67.3 | 2.12 | 0.06 |
| 14 | -27.027833 | -52.633222 | 20.27 | 7.05 | 231 | 82 | 9.0 | 53 | 63.2 | 70.5 | 1.65 | 0.10 |
| 15 | -26.991361 | -52.652361 | 25.29 | 6.77 | 193 | 110 | 0.9 | 72 | 55.2 | 98.4 | 0.21 | 0.11 |
| 16 | -26.991417 | -52.652389 | 20.01 | 5.97 | 323 | 91 | 2.3 | 59 | 65.0 | 79.6 | 4.11 | 0.14 |
| 17 | -26.999611 | -52.614556 | 21.70 | 6.48 | 254 | 98 | 10.1 | 64 | 70.3 | 84.3 | 1.85 | 0.91 |
| 18 | -27.00475 | -52.61675 | 21.46 | 6.11 | 326 | 91 | 7.0 | 59 | 68.6 | 78.2 | 1.69 | 0.02 |
| 19 | -27.027167 | -52.617944 | 20.57 | 6.33 | 250 | 94 | 41.3 | 61 | 51.0 | 80.0 | 1.28 | 0.13 |
| 20 | -27.049306 | -52.624667 | 20.68 | 6.38 | 316 | 134 | 1.4 | 87 | 63.8 | 116.6 | 4.12 | 0.29 |
| 21 | -27.118722 | -52.668333 | 25.19 | 6.54 | 270 | 542 | 3.1 | 347 | 74.2 | 465.0 | 5.29 | 1.13 |
| 22 | -27.121444 | -52.685083 | 22.73 | 7.57 | 142 | 726 | 9.4 | 465 | 64.8 | 598.0 | 5.69 | 1.87 |
| 23 | -27.111444 | -52.660472 | 23.12 | 7.59 | 230 | 272 | 5.9 | 177 | 82.2 | 231.7 | 4.92 | 1.99 |
| 24 | -27.095889 | -52.656806 | 23.46 | 7.10 | 155 | 84 | 1.0 | 55 | 50.2 | 71.0 | 2.88 | 0.23 |
| 25 | -27.095861 | -52.657278 | 24.15 | 6.66 | 271 | 82 | 2.3 | 53 | 53.2 | 69.8 | 1.99 | 0.17 |
| 26 | -27.09419 | -52.658806 | 24.91 | 5.88 | 269 | 83 | 15.5 | 54 | 37.3 | 70.9 | 0.94 | 0.36 |
| 27 | -27.092639 | -52.655333 | 23.81 | 5.58 | 330 | 79 | 9.1 | 51 | 61.7 | 67.7 | 1.95 | 0.14 |
| 28 | -27.027846 | -52.645552 | 19.77 | 6.54 | 301 | 81 | 5.1 | 53 | 72.8 | 69.5 | 2.69 | 0.07 |
| 29 | -27.090415 | -52.644533 | 21.50 | 5.63 | 325 | 1 | 8.8 | 10 | 76.7 | 33.4 | 1.54 | 0.08 |
| 30 | -27.089614 | -52.666751 | 21.52 | 5.94 | 331 | 122 | 0.9 | 80 | 77.5 | 104 | 5.34 | 0.06 |

**Table SM2.** Average values of the sediment variables evaluated along the watershed basin.

| Site | silt + clay | fine sand | medium sand | coarse sand | very coarse sand | pebble | stones |
| --- | --- | --- | --- | --- | --- | --- | --- |
| 1 | 1.6 | 5.2 | 18.0 | 37.1 | 10.8 | 24.2 | 3.1 |
| 2 | 12.8 | 5.5 | 15.3 | 39.3 | 11.1 | 9.7 | 6.4 |
| 3 | 24.3 | 14.3 | 20.1 | 23.3 | 4.2 | 6.9 | 6.9 |
| 4 | 55.8 | 13.4 | 13.8 | 12.4 | 2.9 | 0.9 | 0.9 |
| 5 | 3.3 | 3.6 | 16.7 | 68.3 | 3.9 | 2.1 | 2.1 |
| 6 | 3.3 | 3.6 | 16.7 | 68.3 | 3.9 | 2.1 | 2.1 |
| 7 | 72.3 | 7.2 | 6.1 | 9.4 | 2.7 | 0.4 | 1.9 |
| 8 | 23.0 | 10.2 | 19.4 | 34.7 | 4.8 | 0.7 | 7.2 |
| 9 | 5.8 | 6.8 | 20.8 | 54.1 | 8.2 | 2.2 | 2.2 |
| 10 | 2.3 | 1.4 | 2.9 | 36.6 | 20.8 | 20.4 | 15.6 |
| 11 | 26.9 | 4.4 | 8.9 | 43.3 | 8.8 | 4.4 | 3.3 |
| 12 | 75.6 | 5.6 | 6.7 | 9.0 | 1.2 | 0.8 | 1.1 |
| 13 | 5.7 | 7.3 | 24.0 | 58.4 | 3.2 | 1.0 | 0.4 |
| 14 | 5.7 | 7.3 | 24.0 | 58.4 | 3.2 | 1.0 | 0.4 |
| 15 | 16.3 | 11.0 | 15.6 | 24.2 | 8.2 | 10.1 | 14.8 |
| 16 | 59.0 | 4.3 | 6.5 | 23.0 | 3.7 | 1.0 | 2.6 |
| 17 | 47.7 | 5.5 | 9.6 | 27.6 | 5.8 | 3.5 | 0.3 |
| 18 | 1.1 | 4.3 | 23.0 | 63.8 | 3.4 | 2.9 | 1.5 |
| 19 | 47.7 | 5.5 | 9.6 | 27.6 | 5.8 | 3.5 | 0.3 |
| 20 | 1.1 | 4.3 | 23.0 | 63.8 | 3.4 | 2.9 | 1.5 |
| 21 | 83.2 | 4.7 | 4.8 | 6.8 | 0.3 | 0.1 | 0.1 |
| 22 | 53.2 | 7.8 | 17.1 | 21.2 | 0.5 | 0.1 | 0.1 |
| 23 | 67.3 | 9.9 | 13.3 | 8.7 | 0.4 | 0.2 | 0.2 |
| 24 | 87.4 | 5.3 | 4.8 | 2.3 | 0.1 | 0.1 | 0.1 |
| 25 | 73.5 | 3.3 | 2.7 | 6.1 | 5.4 | 4.2 | 4.8 |
| 26 | 25.8 | 15.0 | 20.2 | 29.3 | 4.1 | 2.6 | 2.9 |
| 27 | 25.8 | 15.0 | 20.2 | 29.3 | 4.1 | 2.6 | 2.9 |
| 28 | 28.3 | 6.0 | 10.4 | 48.3 | 4.4 | 1.2 | 1.4 |
| 29 | 4.3 | 9.5 | 22.8 | 40.7 | 6.6 | 14.7 | 1.4 |
| 30 | 44.3 | 19.0 | 22.8 | 12.5 | 0.5 | 0.2 | 0.6 |

**Table SM3.** The presence or absence of benthic macroinvertebrates and the BMWP index were evaluated along the watershed basin.

| Point | 1 | 2 | 3 | 4 | 5 | 6 | 7 | 8 | 9 | 10 | 11 | 12 | 13 | 14 | 15 | 16 | 17 | 18 | 19 | 20 | 21 | 22 | 23 | 24 | 25 | 26 | 27 | 28 | 29 | 30 |
| --- | --- | --- | --- | --- | --- | --- | --- | --- | --- | --- | --- | --- | --- | --- | --- | --- | --- | --- | --- | --- | --- | --- | --- | --- | --- | --- | --- | --- | --- | --- |
| Veliidae | 0 | 0 | 0 | 0 | 0 | 0 | 0 | 0 | 0 | 0 | 0 | 0 | 0 | 0 | 0 | 0 | 1 | 0 | 0 | 0 | 0 | 0 | 0 | 2 | 0 | 2 | 0 | 0 | 0 | 0 |
| Tricladida | 0 | 0 | 0 | 11 | 1 | 0 | 0 | 0 | 0 | 6 | 0 | 0 | 0 | 1 | 0 | 0 | 0 | 0 | 0 | 0 | 0 | 0 | 0 | 0 | 0 | 1 | 0 | 0 | 0 | 0 |
| Tipulidae | 0 | 0 | 0 | 0 | 0 | 0 | 0 | 1 | 0 | 0 | 0 | 3 | 0 | 0 | 1 | 0 | 1 | 0 | 0 | 0 | 0 | 0 | 1 | 2 | 0 | 1 | 0 | 2 | 1 | 0 |
| Tabanidae | 0 | 0 | 0 | 0 | 0 | 0 | 0 | 1 | 0 | 0 | 0 | 0 | 0 | 0 | 0 | 0 | 0 | 0 | 0 | 0 | 0 | 0 | 0 | 0 | 0 | 0 | 1 | 0 | 1 | 0 |
| Stratyiomyidae | 0 | 0 | 0 | 0 | 0 | 0 | 0 | 0 | 0 | 0 | 0 | 0 | 0 | 0 | 0 | 0 | 0 | 0 | 0 | 0 | 0 | 2 | 0 | 0 | 0 | 0 | 0 | 0 | 0 | 0 |
| Staphylinidae | 0 | 0 | 0 | 0 | 0 | 0 | 0 | 0 | 0 | 0 | 0 | 0 | 0 | 0 | 1 | 0 | 0 | 1 | 0 | 0 | 1 | 0 | 0 | 0 | 0 | 0 | 0 | 0 | 0 | 0 |
| Simulidae | 13 | 2 | 0 | 76 | 3 | 1 | 0 | 1 | 31 | 0 | 21 | 3 | 1 | 3 | 0 | 0 | 3 | 4 | 2 | 3 | 1 | 2 | 4 | 0 | 0 | 20 | 36 | 11 | 69 | 4 |
| Scirtidae | 0 | 0 | 0 | 0 | 0 | 0 | 0 | 0 | 0 | 0 | 0 | 0 | 0 | 0 | 1 | 0 | 0 | 0 | 0 | 0 | 0 | 0 | 0 | 0 | 0 | 1 | 0 | 0 | 0 | 0 |
| Psychodidae | 0 | 0 | 0 | 0 | 0 | 0 | 0 | 0 | 0 | 0 | 0 | 0 | 0 | 0 | 0 | 0 | 0 | 0 | 2 | 0 | 0 | 0 | 0 | 0 | 0 | 0 | 0 | 0 | 0 | 0 |
| Pyralidae | 0 | 0 | 0 | 0 | 0 | 0 | 0 | 0 | 0 | 0 | 0 | 0 | 1 | 0 | 0 | 0 | 0 | 0 | 0 | 0 | 0 | 0 | 0 | 0 | 0 | 0 | 0 | 0 | 0 | 0 |
| Polycentropodidae | 0 | 0 | 0 | 0 | 0 | 0 | 0 | 0 | 0 | 0 | 0 | 0 | 0 | 0 | 0 | 0 | 0 | 0 | 0 | 0 | 0 | 0 | 0 | 0 | 0 | 1 | 0 | 7 | 0 | 0 |
| Planorbidae | 2 | 0 | 0 | 2 | 0 | 1 | 0 | 1 | 1 | 0 | 0 | 0 | 0 | 2 | 1 | 4 | 0 | 0 | 9 | 0 | 0 | 0 | 0 | 0 | 0 | 0 | 0 | 0 | 0 | 1 |
| Physidae | 1 | 0 | 2 | 0 | 0 | 0 | 0 | 0 | 0 | 0 | 3 | 0 | 0 | 2 | 45 | 0 | 4 | 2 | 0 | 0 | 1 | 0 | 0 | 1 | 0 | 0 | 1 | 0 | 0 | 0 |
| Philosciidae | 0 | 0 | 0 | 0 | 0 | 0 | 0 | 1 | 0 | 0 | 0 | 1 | 0 | 0 | 2 | 0 | 0 | 0 | 0 | 0 | 0 | 0 | 0 | 0 | 0 | 0 | 0 | 0 | 0 | 0 |
| Philopotamidae | 0 | 0 | 0 | 0 | 0 | 0 | 0 | 0 | 0 | 1 | 0 | 0 | 1 | 0 | 0 | 0 | 0 | 0 | 0 | 0 | 0 | 0 | 0 | 0 | 0 | 0 | 1 | 2 | 0 | 0 |
| Ostracoda | 0 | 0 | 0 | 0 | 0 | 0 | 0 | 0 | 1 | 0 | 0 | 0 | 0 | 0 | 1 | 0 | 0 | 0 | 0 | 0 | 0 | 0 | 0 | 0 | 0 | 0 | 0 | 0 | 1 | 0 |
| Oligochaeta | 2 | 0 | 0 | 32 | 1 | 1 | 3 | 6 | 4 | 2 | 1 | 0 | 10 | 12 | 55 | 5 | 1 | 28 | 6 | 3 | 0 | 15 | 5 | 2 | 1 | 3 | 6 | 1 | 1 | 13 |
| Odontoceridae | 0 | 1 | 0 | 0 | 1 | 0 | 0 | 0 | 0 | 0 | 0 | 0 | 0 | 0 | 0 | 0 | 0 | 0 | 0 | 0 | 0 | 0 | 0 | 0 | 0 | 0 | 0 | 0 | 0 | 0 |
| Notonectidae | 0 | 0 | 0 | 2 | 0 | 0 | 0 | 1 | 0 | 0 | 0 | 0 | 0 | 4 | 0 | 0 | 0 | 0 | 0 | 0 | 0 | 0 | 0 | 0 | 3 | 45 | 1 | 0 | 0 | 0 |
| Mesoveliidae | 0 | 0 | 0 | 1 | 0 | 0 | 0 | 0 | 0 | 0 | 0 | 0 | 0 | 0 | 0 | 0 | 0 | 0 | 0 | 0 | 0 | 0 | 0 | 0 | 0 | 0 | 1 | 0 | 1 | 0 |
| Lymnaeidae | 0 | 0 | 0 | 0 | 0 | 0 | 0 | 0 | 0 | 0 | 0 | 0 | 0 | 0 | 2 | 0 | 2 | 0 | 0 | 0 | 0 | 0 | 0 | 0 | 0 | 0 | 0 | 0 | 0 | 0 |
| Libellulidae | 0 | 8 | 0 | 0 | 1 | 1 | 0 | 0 | 0 | 0 | 8 | 1 | 2 | 3 | 1 | 6 | 4 | 0 | 1 | 0 | 1 | 2 | 5 | 1 | 0 | 3 | 0 | 5 | 1 | 0 |
| Leptophlebiidae | 0 | 0 | 0 | 0 | 1 | 18 | 0 | 4 | 5 | 0 | 0 | 3 | 0 | 0 | 1 | 0 | 0 | 1 | 0 | 0 | 0 | 0 | 0 | 9 | 0 | 20 | 0 | 20 | 0 | 0 |
| Leptohyphidae | 0 | 0 | 0 | 0 | 0 | 0 | 0 | 0 | 0 | 0 | 0 | 0 | 1 | 0 | 0 | 0 | 0 | 0 | 0 | 0 | 0 | 0 | 0 | 1 | 0 | 0 | 0 | 0 | 0 | 0 |
| Hydropsychidae | 16 | 84 | 6 | 1 | 6 | 28 | 2 | 1 | 1 | 4 | 65 | 29 | 15 | 9 | 0 | 107 | 9 | 103 | 1 | 1 | 33 | 38 | 38 | 5 | 0 | 4 | 47 | 12 | 4 | 7 |
| Hydrophilidae | 0 | 0 | 0 | 0 | 0 | 0 | 0 | 0 | 9 | 0 | 0 | 0 | 2 | 0 | 0 | 0 | 0 | 0 | 0 | 0 | 0 | 0 | 0 | 1 | 0 | 0 | 0 | 3 | 0 | 0 |
| Hydrobiidae | 0 | 0 | 0 | 0 | 0 | 0 | 0 | 0 | 0 | 0 | 0 | 0 | 0 | 0 | 0 | 1 | 0 | 0 | 0 | 0 | 0 | 0 | 0 | 0 | 0 | 0 | 13 | 0 | 0 | 0 |
| Hydrobiosidae | 0 | 0 | 0 | 0 | 0 | 0 | 0 | 0 | 0 | 0 | 1 | 0 | 0 | 0 | 0 | 0 | 0 | 4 | 0 | 0 | 0 | 0 | 0 | 0 | 0 | 0 | 0 | 0 | 0 | 0 |
| Hydracarina | 0 | 0 | 0 | 0 | 0 | 0 | 0 | 0 | 0 | 0 | 0 | 0 | 0 | 0 | 0 | 0 | 1 | 0 | 0 | 0 | 0 | 0 | 0 | 0 | 0 | 0 | 0 | 0 | 0 | 0 |
| Hyalellidae | 0 | 0 | 0 | 0 | 0 | 0 | 0 | 0 | 0 | 0 | 0 | 0 | 0 | 1 | 0 | 0 | 0 | 0 | 0 | 1 | 0 | 0 | 0 | 0 | 41 | 81 | 2 | 0 | 4 | 0 |
| Hirudinidae | 3 | 5 | 12 | 78 | 5 | 1 | 5 | 5 | 2 | 0 | 3 | 4 | 0 | 3 | 157 | 5 | 7 | 3 | 2 | 25 | 12 | 17 | 9 | 6 | 0 | 4 | 2 | 0 | 5 | 46 |
| Hebridae | 0 | 0 | 1 | 1 | 0 | 0 | 0 | 0 | 0 | 0 | 0 | 0 | 0 | 0 | 0 | 0 | 0 | 3 | 1 | 0 | 0 | 0 | 0 | 0 | 0 | 0 | 2 | 0 | 0 | 0 |
| Gyrinidae | 0 | 4 | 1 | 0 | 0 | 1 | 0 | 0 | 9 | 0 | 0 | 0 | 3 | 0 | 0 | 1 | 1 | 2 | 0 | 0 | 2 | 13 | 3 | 5 | 0 | 0 | 0 | 1 | 1 | 2 |
| Gomphidae | 0 | 0 | 0 | 0 | 1 | 0 | 0 | 0 | 0 | 0 | 0 | 0 | 0 | 0 | 0 | 0 | 0 | 0 | 0 | 0 | 0 | 1 | 0 | 0 | 0 | 0 | 0 | 0 | 0 | 0 |
| Glipopterigydae | 0 | 0 | 0 | 0 | 0 | 0 | 0 | 0 | 0 | 0 | 0 | 0 | 0 | 0 | 0 | 0 | 0 | 0 | 0 | 0 | 0 | 0 | 0 | 0 | 0 | 0 | 0 | 1 | 0 | 0 |
| Gerridae | 0 | 0 | 0 | 0 | 0 | 0 | 0 | 0 | 0 | 0 | 0 | 0 | 0 | 0 | 2 | 0 | 1 | 0 | 0 | 0 | 0 | 0 | 0 | 0 | 0 | 2 | 0 | 1 | 0 | 5 |
| Empididae | 0 | 0 | 0 | 0 | 0 | 0 | 0 | 0 | 0 | 0 | 0 | 0 | 0 | 0 | 0 | 0 | 0 | 0 | 0 | 0 | 0 | 0 | 0 | 0 | 0 | 0 | 0 | 0 | 10 | 0 |
| Elmidae | 3 | 3 | 0 | 0 | 0 | 10 | 2 | 8 | 3 | 0 | 18 | 2 | 7 | 1 | 1 | 0 | 6 | 2 | 0 | 0 | 0 | 1 | 0 | 9 | 2 | 6 | 1 | 10 | 7 | 0 |
| Dytiscidae | 0 | 0 | 0 | 1 | 2 | 2 | 0 | 0 | 0 | 1 | 0 | 0 | 0 | 1 | 0 | 0 | 0 | 0 | 0 | 0 | 0 | 0 | 0 | 0 | 0 | 0 | 0 | 0 | 0 | 0 |
| Culicidae | 0 | 0 | 0 | 0 | 0 | 1 | 0 | 0 | 2 | 0 | 0 | 0 | 0 | 1 | 0 | 1 | 0 | 13 | 0 | 0 | 0 | 0 | 0 | 0 | 0 | 0 | 28 | 0 | 3 | 0 |
| Corydalidae | 0 | 0 | 0 | 0 | 0 | 0 | 0 | 0 | 0 | 0 | 0 | 0 | 0 | 0 | 0 | 0 | 0 | 0 | 0 | 0 | 0 | 0 | 0 | 0 | 0 | 0 | 0 | 2 | 0 | 0 |
| Corixidae | 0 | 8 | 2 | 0 | 0 | 0 | 0 | 0 | 0 | 0 | 0 | 0 | 3 | 0 | 0 | 0 | 0 | 0 | 0 | 1 | 0 | 0 | 0 | 0 | 0 | 1 | 0 | 0 | 0 | 0 |
| Collembola | 0 | 0 | 0 | 3 | 5 | 0 | 0 | 0 | 0 | 1 | 0 | 0 | 0 | 3 | 0 | 1 | 0 | 4 | 0 | 2 | 1 | 1 | 0 | 2 | 3 | 6 | 5 | 4 | 0 | 0 |
| Coenagrionidae | 0 | 0 | 0 | 0 | 1 | 0 | 0 | 1 | 4 | 0 | 0 | 0 | 0 | 0 | 0 | 1 | 1 | 2 | 4 | 0 | 21 | 0 | 0 | 0 | 0 | 0 | 0 | 0 | 0 | 2 |
| Chironomidae | 18 | 112 | 89 | 110 | 67 | 40 | 9 | 21 | 116 | 9 | 120 | 58 | 185 | 94 | 58 | 88 | 43 | 52 | 15 | 69 | 66 | 526 | 14 | 64 | 15 | 98 | 93 | 41 | 224 | 120 |
| Ceratopogonidae | 0 | 6 | 98 | 1 | 0 | 0 | 1 | 0 | 0 | 1 | 3 | 0 | 0 | 0 | 0 | 0 | 0 | 0 | 2 | 0 | 0 | 0 | 23 | 2 | 1 | 1 | 2 | 2 | 1 | 0 |
| Calopterygidae | 0 | 5 | 1 | 2 | 1 | 2 | 2 | 1 | 5 | 1 | 4 | 4 | 7 | 16 | 1 | 0 | 0 | 3 | 6 | 6 | 5 | 11 | 0 | 2 | 0 | 7 | 0 | 3 | 17 | 3 |
| Calamoceratidae | 3 | 1 | 0 | 0 | 9 | 2 | 1 | 1 | 11 | 0 | 21 | 3 | 0 | 0 | 0 | 1 | 0 | 0 | 0 | 1 | 0 | 2 | 2 | 1 | 0 | 7 | 4 | 0 | 0 | 0 |
| Caenidae | 2 | 2 | 0 | 0 | 6 | 5 | 4 | 9 | 24 | 1 | 0 | 5 | 5 | 0 | 1 | 0 | 0 | 0 | 1 | 3 | 0 | 2 | 0 | 5 | 0 | 2 | 3 | 3 | 0 | 1 |
| Bivalvia | 0 | 0 | 0 | 25 | 0 | 4 | 0 | 5 | 0 | 0 | 0 | 0 | 1 | 0 | 0 | 0 | 0 | 2 | 0 | 0 | 0 | 0 | 0 | 0 | 0 | 0 | 0 | 0 | 0 | 0 |
| Belostomatidae | 0 | 0 | 0 | 4 | 4 | 0 | 0 | 0 | 0 | 0 | 0 | 0 | 0 | 0 | 2 | 0 | 0 | 2 | 0 | 0 | 0 | 0 | 0 | 1 | 0 | 3 | 0 | 0 | 0 | 0 |
| Baetidae | 45 | 52 | 0 | 6 | 29 | 93 | 4 | 44 | 271 | 4 | 0 | 3 | 36 | 26 | 3 | 25 | 1 | 26 | 2 | 8 | 1 | 7 | 3 | 39 | 5 | 6 | 2 | 7 | 7 | 3 |
| Ancylidae | 0 | 0 | 0 | 0 | 0 | 0 | 0 | 0 | 0 | 0 | 0 | 0 | 0 | 0 | 0 | 0 | 1 | 0 | 0 | 0 | 0 | 0 | 0 | 3 | 1 | 1 | 0 | 0 | 0 | 0 |
| Aeglidae | 5 | 6 | 0 | 0 | 0 | 4 | 18 | 0 | 0 | 0 | 0 | 0 | 0 | 0 | 0 | 0 | 0 | 0 | 0 | 0 | 0 | 0 | 0 | 0 | 0 | 0 | 0 | 0 | 0 | 0 |
| Amphipoda | 0 | 0 | 0 | 0 | 0 | 0 | 0 | 0 | 0 | 0 | 1 | 0 | 0 | 0 | 0 | 0 | 0 | 0 | 0 | 0 | 0 | 0 | 0 | 0 | 0 | 0 | 0 | 0 | 0 | 0 |
| Aeshnidae | 2 | 0 | 0 | 0 | 0 | 0 | 1 | 0 | 0 | 0 | 1 | 0 | 0 | 0 | 1 | 0 | 0 | 0 | 0 | 0 | 0 | 0 | 0 | 0 | 0 | 0 | 0 | 0 | 0 | 1 |
| Taxa_present | 13 | 15 | 9 | 17 | 18 | 18 | 12 | 18 | 17 | 11 | 14 | 13 | 16 | 17 | 20 | 13 | 17 | 19 | 14 | 12 | 12 | 15 | 11 | 21 | 9 | 25 | 20 | 20 | 18 | 13 |
| BMWP | 50 | 73 | 17 | 75 | 86 | 83 | 32 | 69 | 79 | 34 | 46 | 27 | 49 | 45 | 45 | 37 | 31 | 61 | 46 | 36 | 14 | 31 | 35 | 85 | 21 | 98 | 48 | 30 | 26 | 44 |
| ASPT | 5.0 | 5.2 | 5.7 | 6.3 | 6.1 | 5.5 | 5.3 | 5.8 | 5.3 | 5.7 | 5.8 | 3.9 | 5.4 | 5.6 | 6.4 | 5.3 | 6.2 | 6.1 | 6.6 | 5.1 | 7.0 | 5.2 | 5.8 | 6.1 | 4.2 | 5.8 | 5.3 | 6.0 | 5.2 | 6.3 |

**Table** **SM4.** Presence or absence of phytoplankton along the watershed basin.

|  | Site | | | | | | | | | | | | | | | | | | | | | | | | | | | | | |
| --- | --- | --- | --- | --- | --- | --- | --- | --- | --- | --- | --- | --- | --- | --- | --- | --- | --- | --- | --- | --- | --- | --- | --- | --- | --- | --- | --- | --- | --- | --- |
|  | 1 | 2 | 3 | 4 | 5 | 6 | 7 | 8 | 9 | 10 | 11 | 12 | 13 | 14 | 15 | 16 | 17 | 18 | 19 | 20 | 21 | 22 | 23 | 24 | 25 | 26 | 27 | 28 | 29 | 30 |
| Closterium | 1 | 1 | 1 | 1 | 1 | 1 | 1 | 1 | 1 | 1 | 0 | 1 | 0 | 1 | 1 | 1 | 1 | 0 | 0 | 1 | 1 | 0 | 0 | 0 | 1 | 1 | 1 | 1 | 1 | 1 |
| Coelastrum | 0 | 0 | 0 | 1 | 0 | 0 | 1 | 1 | 0 | 1 | 0 | 1 | 0 | 0 | 1 | 1 | 0 | 1 | 1 | 1 | 0 | 0 | 1 | 1 | 1 | 1 | 0 | 0 | 0 | 0 |
| Cosmarium | 0 | 0 | 0 | 0 | 0 | 0 | 0 | 0 | 0 | 0 | 0 | 0 | 0 | 0 | 1 | 0 | 0 | 0 | 1 | 0 | 0 | 1 | 1 | 0 | 0 | 0 | 0 | 0 | 0 | 0 |
| Crucigenia | 0 | 0 | 0 | 0 | 1 | 0 | 0 | 0 | 0 | 0 | 0 | 1 | 0 | 0 | 0 | 1 | 0 | 1 | 0 | 0 | 0 | 0 | 0 | 0 | 0 | 0 | 0 | 0 | 0 | 0 |
| Desmodesmus | 0 | 1 | 1 | 0 | 1 | 0 | 0 | 0 | 1 | 1 | 1 | 1 | 1 | 0 | 1 | 1 | 0 | 1 | 0 | 0 | 1 | 0 | 1 | 1 | 1 | 1 | 1 | 1 | 1 | 0 |
| Euastrum | 0 | 0 | 0 | 1 | 0 | 0 | 0 | 0 | 0 | 0 | 0 | 0 | 0 | 0 | 0 | 1 | 0 | 0 | 1 | 0 | 1 | 0 | 0 | 0 | 0 | 1 | 0 | 1 | 1 | 0 |
| Euglena | 0 | 1 | 0 | 0 | 0 | 1 | 0 | 0 | 0 | 1 | 0 | 0 | 1 | 0 | 0 | 0 | 0 | 0 | 0 | 0 | 1 | 0 | 1 | 1 | 1 | 1 | 0 | 1 | 0 | 0 |
| Gyrosigma | 0 | 0 | 0 | 1 | 1 | 1 | 1 | 0 | 1 | 0 | 0 | 1 | 1 | 1 | 0 | 0 | 3 | 0 | 0 | 1 | 1 | 0 | 1 | 0 | 0 | 0 | 1 | 1 | 1 | 0 |
| Hariotina | 0 | 0 | 0 | 0 | 0 | 1 | 0 | 0 | 0 | 0 | 0 | 0 | 0 | 0 | 1 | 0 | 0 | 0 | 0 | 0 | 0 | 1 | 0 | 0 | 0 | 0 | 0 | 0 | 1 | 0 |
| Kirchneriella | 0 | 0 | 0 | 0 | 0 | 0 | 0 | 0 | 0 | 1 | 0 | 1 | 0 | 0 | 0 | 1 | 0 | 1 | 0 | 0 | 0 | 1 | 0 | 1 | 1 | 0 | 0 | 1 | 0 | 1 |
| Komvophoron | 0 | 0 | 0 | 0 | 0 | 0 | 0 | 0 | 0 | 0 | 1 | 1 | 0 | 0 | 0 | 0 | 1 | 0 | 0 | 0 | 0 | 0 | 0 | 0 | 0 | 0 | 0 | 0 | 0 | 0 |
| Micrasterias | 0 | 0 | 0 | 0 | 0 | 0 | 0 | 0 | 1 | 1 | 0 | 0 | 0 | 0 | 0 | 0 | 0 | 0 | 0 | 0 | 0 | 0 | 0 | 0 | 0 | 1 | 0 | 0 | 0 | 0 |
| Pediastrum | 1 | 0 | 0 | 1 | 0 | 1 | 0 | 1 | 1 | 1 | 0 | 1 | 0 | 1 | 1 | 1 | 0 | 1 | 0 | 0 | 1 | 0 | 0 | 1 | 1 | 0 | 1 | 1 | 0 | 0 |
| Scenedesmus | 1 | 0 | 0 | 1 | 0 | 0 | 1 | 1 | 0 | 1 | 1 | 1 | 0 | 0 | 1 | 0 | 0 | 1 | 0 | 0 | 1 | 0 | 0 | 1 | 1 | 1 | 1 | 0 | 0 | 0 |
| Spirogyra | 0 | 0 | 0 | 0 | 0 | 0 | 0 | 0 | 0 | 0 | 0 | 0 | 0 | 0 | 0 | 0 | 0 | 0 | 0 | 0 | 0 | 0 | 0 | 0 | 0 | 0 | 0 | 1 | 1 | 1 |
| Spirulina | 0 | 1 | 0 | 1 | 0 | 0 | 0 | 1 | 0 | 1 | 0 | 0 | 0 | 0 | 0 | 0 | 0 | 0 | 0 | 0 | 1 | 0 | 0 | 0 | 0 | 0 | 0 | 1 | 0 | 0 |
| Staurastrum | 0 | 0 | 0 | 1 | 0 | 0 | 0 | 0 | 0 | 0 | 0 | 0 | 0 | 0 | 0 | 0 | 0 | 1 | 0 | 0 | 0 | 0 | 0 | 0 | 0 | 1 | 0 | 1 | 0 | 0 |
| Stauridium | 0 | 1 | 0 | 0 | 0 | 0 | 0 | 0 | 0 | 0 | 0 | 0 | 0 | 0 | 0 | 0 | 0 | 0 | 0 | 0 | 0 | 0 | 0 | 1 | 0 | 0 | 1 | 0 | 0 | 0 |
| Surirella | 1 | 1 | 1 | 1 | 1 | 1 | 1 | 1 | 1 | 0 | 0 | 1 | 0 | 1 | 1 | 1 | 1 | 1 | 1 | 1 | 0 | 0 | 1 | 0 | 0 | 0 | 1 | 1 | 0 | 1 |
| Tetraëdriella | 0 | 0 | 0 | 0 | 0 | 0 | 0 | 0 | 0 | 0 | 1 | 1 | 1 | 0 | 0 | 0 | 0 | 0 | 0 | 0 | 0 | 0 | 0 | 0 | 0 | 0 | 0 | 0 | 0 | 0 |
| Tetraedron | 0 | 0 | 0 | 0 | 0 | 0 | 0 | 0 | 0 | 0 | 0 | 0 | 0 | 0 | 0 | 0 | 0 | 0 | 0 | 0 | 0 | 1 | 1 | 0 | 0 | 1 | 0 | 0 | 0 | 0 |
| Tetrastrum | 0 | 0 | 0 | 0 | 0 | 0 | 0 | 0 | 0 | 0 | 0 | 0 | 0 | 0 | 1 | 0 | 0 | 0 | 0 | 0 | 0 | 1 | 0 | 0 | 1 | 0 | 0 | 0 | 0 | 0 |
| Treubaria | 0 | 0 | 0 | 0 | 0 | 0 | 1 | 0 | 1 | 0 | 1 | 0 | 0 | 1 | 0 | 0 | 0 | 0 | 1 | 0 | 0 | 0 | 0 | 0 | 0 | 0 | 0 | 0 | 0 | 0 |
| Tropidoscyphus | 0 | 0 | 0 | 0 | 0 | 0 | 0 | 0 | 0 | 1 | 0 | 0 | 1 | 0 | 0 | 0 | 0 | 0 | 0 | 0 | 0 | 1 | 0 | 0 | 0 | 0 | 0 | 1 | 0 | 0 |
| Westella | 0 | 1 | 0 | 0 | 0 | 0 | 0 | 0 | 0 | 0 | 0 | 0 | 0 | 0 | 0 | 0 | 0 | 0 | 0 | 0 | 0 | 1 | 1 | 1 | 0 | 0 | 0 | 0 | 1 | 0 |

**Table SM5.** Settings and statistics for the reduced Random Forest and BRT models. tc = tree complexity; lr = learning rate; na = not applicable; CV = cross-validation (a).

|  | **Macroinvertebrates** | **BMWP** | **Phytoplankton** |
| --- | --- | --- | --- |
| **Reduced Random Forest and BRT models** | |  |  |
| Data transformation | na | na | na |
| Model error | Gaussian | Gaussian | Gaussian |
| tc | 5 | 5 | 5 |
| lr | 0.001 | 0.001 | 0.001 |
| Total Deviance | 2235.9 | 73355 | 0.021 |
| Residual Deviance | 2085.0 | 52228 | 4.75 |
| Variance Explained (%) | 17.23 | 37.89 | 14.21 |
| Training data correlation | 0.47 | 0.65 | 0.45 |
| CV Deviance (mean) | 39.92 | 338.0 | 0.026 |
| CV Deviance (se) | 17.43 | 37.9 | 0.011 |
| CV correlation | 0.12 | 0.49 | 0.19 |
| CV correlation (se) | 0.02 | 0.06 | 0.01 |

**Table SM6.** Results of the Threshold Indicator Taxa Analysis (TITAN) of the response of the macroinvertebrate community to temperature (a), water electrical conductivity (b), percentage of coarse sand in the sediment stream (c), and percentage of pebbles in the sediment stream (d) along the watershed basin. [freq = number of non-zero abundance values; IndVal = indication value of the taxa; obsiv.prob = probability of an equal or larger IndVal from random permutations; zscore = IndVal expressed as a z-score; 95%, quantile thresholds among bootstrap replicates; purity, proportion of the correct response, whether negative (z - ) or positive (z +); reliability, fidelity of taxa to the group, p ≤ 0.05; z.median = median z-score the across replicates].

|  | **freqthe** | **IndVal** | **obsiv.prob** | **zscore** | **95%** | **purity** | **reliability** | **z.median** |
| --- | --- | --- | --- | --- | --- | --- | --- | --- |
| **(a) Temperature** |  |  |  |  |  |  |  |  |
| aeshnidae | 5.00 | 14.22 | 0.14 | 2.68 | 25.27 | 0.44 | 0.64 | 3.87 |
| aeglidae | 6.00 | 16.20 | 0.02 | 9.63 | 20.57 | 1.00 | 0.98 | 10.10 |
| ancylidae | 4.00 | 6.56 | 0.02 | 4.58 | 24.64 | 0.98 | 0.82 | 6.71 |
| baetidae | 73.00 | 48.60 | 0.02 | 5.80 | 23.56 | 1.00 | 1.00 | 5.16 |
| belostomatidae | 8.00 | 10.29 | 0.04 | 3.22 | 25.22 | 0.98 | 0.86 | 4.25 |
| bivalvia | 6.00 | 13.92 | 0.02 | 7.73 | 21.51 | 0.94 | 0.88 | 9.02 |
| caenidae | 35.00 | 31.99 | 0.04 | 3.08 | 24.32 | 0.74 | 0.84 | 3.82 |
| calamoceratidae | 26.00 | 12.97 | 0.14 | 1.41 | 24.26 | 0.46 | 0.88 | 3.76 |
| calopterygidae | 39.00 | 16.34 | 0.20 | 1.03 | 24.79 | 0.44 | 0.68 | 3.00 |
| ceratopogonidae | 17.00 | 10.83 | 0.18 | 0.98 | 23.31 | 0.56 | 0.36 | 1.78 |
| chironomidae | 142.00 | 68.95 | 0.16 | 1.01 | 24.19 | 0.70 | 0.70 | 2.77 |
| coenagrionidae | 12.00 | 17.32 | 0.02 | 5.14 | 25.19 | 0.74 | 0.64 | 4.08 |
| collembola | 25.00 | 16.12 | 0.02 | 3.63 | 24.64 | 0.92 | 0.78 | 5.04 |
| corixidae | 6.00 | 10.68 | 0.02 | 7.07 | 24.91 | 0.76 | 0.68 | 4.16 |
| culicidae | 10.00 | 6.62 | 0.22 | 0.76 | 23.81 | 0.60 | 0.66 | 3.07 |
| dytiscidae | 6.00 | 15.34 | 0.04 | 2.44 | 22.71 | 0.68 | 0.64 | 3.10 |
| elmidae | 41.00 | 52.78 | 0.02 | 6.00 | 22.25 | 0.96 | 0.88 | 5.78 |
| gerridae | 6.00 | 13.09 | 0.02 | 6.20 | 25.06 | 0.72 | 0.78 | 7.23 |
| gyrinidae | 25.00 | 13.00 | 0.06 | 2.05 | 23.73 | 0.68 | 0.78 | 3.72 |
| hebridae | 5.00 | 3.69 | 0.14 | 1.03 | 23.31 | 0.60 | 0.42 | 2.08 |
| hirudinidae | 70.00 | 95.33 | 0.02 | 10.83 | 25.29 | 1.00 | 1.00 | 6.39 |
| hyalellidae | 7.00 | 11.87 | 0.02 | 6.58 | 24.91 | 0.92 | 0.86 | 6.56 |
| hydrophilidae | 4.00 | 11.28 | 0.02 | 6.02 | 21.20 | 0.98 | 0.90 | 7.34 |
| hydropsychidae | 65.00 | 24.98 | 0.14 | 1.13 | 25.19 | 0.82 | 0.66 | 2.48 |
| leptophlebiidae | 15.00 | 18.10 | 0.02 | 5.25 | 24.91 | 0.46 | 0.98 | 6.97 |
| libellulidae | 30.00 | 19.12 | 0.02 | 3.33 | 24.12 | 0.82 | 0.78 | 4.01 |
| mesoveliidae | 3.00 | 2.63 | 0.32 | 0.97 | 23.81 | 0.72 | 0.20 | 1.99 |
| notonectidae | 11.00 | 17.08 | 0.02 | 6.96 | 24.91 | 1.00 | 0.94 | 8.22 |
| oligochaeta | 49.00 | 36.86 | 0.04 | 2.48 | 25.29 | 0.42 | 0.50 | 2.30 |
| ostracoda | 3.00 | 18.92 | 0.08 | 3.92 | 25.29 | 0.62 | 0.48 | 4.74 |
| philopotamidae | 4.00 | 11.74 | 0.02 | 8.59 | 22.62 | 0.76 | 0.72 | 4.91 |
| philosciidae | 3.00 | 8.29 | 0.02 | 11.00 | 25.24 | 0.94 | 0.68 | 6.62 |
| physidae | 19.00 | 82.26 | 0.02 | 18.47 | 25.29 | 1.00 | 0.96 | 15.64 |
| planorbidae | 12.00 | 13.04 | 0.02 | 6.03 | 25.24 | 0.88 | 1.00 | 6.56 |
| simulidae | 44.00 | 22.18 | 0.04 | 2.73 | 23.73 | 0.70 | 0.84 | 3.18 |
| staphylinidae | 3.00 | 9.41 | 0.02 | 7.95 | 25.29 | 0.84 | 0.68 | 7.75 |
| tabanidae | 3.00 | 3.16 | 0.14 | 2.50 | 23.46 | 0.90 | 0.30 | 2.17 |
| tipulidae | 9.00 | 7.68 | 0.02 | 3.13 | 25.19 | 0.94 | 0.90 | 5.39 |
| tricladida | 5.00 | 3.22 | 0.40 | 0.63 | 24.64 | 0.70 | 0.38 | 1.84 |
| veliidae | 3.00 | 4.31 | 0.02 | 4.57 | 24.91 | 0.96 | 0.60 | 4.68 |
| **(b) Electrical conductivity** | |  |  |  |  |  |  |  |
| aeshnidae | 5.00 | 5.48 | 0.08 | 1.85 | 0.11 | 0.56 | 0.68 | 4.27 |
| aeglidae | 6.00 | 12.77 | 0.02 | 10.01 | 0.08 | 1.00 | 1.00 | 9.57 |
| ancylidae | 4.00 | 3.60 | 0.08 | 1.65 | 0.10 | 0.78 | 0.24 | 1.81 |
| baetidae | 73.00 | 39.10 | 0.02 | 3.10 | 0.11 | 0.98 | 0.96 | 3.61 |
| belostomatidae | 8.00 | 6.67 | 0.04 | 3.50 | 0.10 | 1.00 | 0.78 | 3.60 |
| bivalvia | 6.00 | 7.17 | 0.04 | 2.33 | 0.10 | 0.80 | 0.62 | 4.46 |
| caenidae | 35.00 | 17.05 | 0.06 | 1.65 | 0.11 | 0.74 | 0.92 | 3.59 |
| calamoceratidae | 26.00 | 15.48 | 0.02 | 2.80 | 0.40 | 0.80 | 0.88 | 3.61 |
| calopterygidae | 39.00 | 21.39 | 0.18 | 1.00 | 0.54 | 0.52 | 0.70 | 3.32 |
| ceratopogonidae | 17.00 | 10.00 | 0.52 | 0.86 | 0.24 | 0.60 | 0.62 | 2.69 |
| chironomidae | 142.00 | 86.08 | 0.02 | 4.35 | 0.73 | 0.58 | 0.98 | 4.16 |
| coenagrionidae | 12.00 | 14.52 | 0.04 | 3.60 | 0.54 | 0.98 | 0.92 | 4.80 |
| collembola | 25.00 | 17.12 | 0.02 | 2.92 | 0.20 | 0.64 | 0.94 | 4.10 |
| corixidae | 6.00 | 6.99 | 0.02 | 3.48 | 0.13 | 0.88 | 0.76 | 4.49 |
| culicidae | 10.00 | 9.26 | 0.02 | 3.42 | 0.10 | 0.98 | 0.88 | 4.92 |
| dytiscidae | 6.00 | 4.26 | 0.08 | 0.89 | 0.10 | 0.48 | 0.48 | 2.50 |
| elmidae | 41.00 | 27.69 | 0.02 | 8.33 | 0.12 | 0.98 | 1.00 | 7.93 |
| gerridae | 6.00 | 4.41 | 0.18 | 1.42 | 0.11 | 0.74 | 0.38 | 2.44 |
| gyrinidae | 25.00 | 60.86 | 0.02 | 11.00 | 0.73 | 0.98 | 1.00 | 9.14 |
| hebridae | 5.00 | 4.46 | 0.02 | 1.93 | 0.10 | 0.60 | 0.28 | 2.11 |
| hirudinidae | 70.00 | 46.31 | 0.02 | 7.55 | 0.11 | 1.00 | 1.00 | 7.41 |
| hyalellidae | 7.00 | 10.53 | 0.04 | 5.75 | 0.10 | 1.00 | 0.86 | 4.09 |
| hydrophilidae | 4.00 | 2.94 | 0.30 | 0.68 | 0.10 | 0.74 | 0.34 | 2.08 |
| hydropsychidae | 65.00 | 36.62 | 0.06 | 2.09 | 0.73 | 0.62 | 0.72 | 2.94 |
| leptophlebiidae | 15.00 | 10.73 | 0.02 | 3.59 | 0.10 | 0.94 | 0.90 | 3.78 |
| libellulidae | 30.00 | 38.64 | 0.02 | 4.44 | 0.27 | 0.54 | 0.88 | 4.79 |
| mesoveliidae | 3.00 | 4.51 | 0.06 | 4.41 | 0.10 | 0.82 | 0.56 | 4.85 |
| notonectidae | 11.00 | 12.02 | 0.02 | 4.93 | 0.10 | 0.98 | 0.98 | 4.48 |
| oligochaeta | 49.00 | 25.24 | 0.12 | 1.58 | 0.73 | 0.80 | 0.56 | 2.42 |
| ostracoda | 3.00 | 15.59 | 0.06 | 4.85 | 0.10 | 0.70 | 0.68 | 6.09 |
| philopotamidae | 4.00 | 4.81 | 0.02 | 3.11 | 0.10 | 0.94 | 0.74 | 4.26 |
| philosciidae | 3.00 | 2.86 | 0.10 | 1.37 | 0.11 | 0.48 | 0.44 | 2.77 |
| physidae | 19.00 | 18.10 | 0.02 | 4.87 | 0.11 | 0.96 | 0.88 | 4.42 |
| planorbidae | 12.00 | 6.38 | 0.14 | 1.47 | 0.11 | 0.58 | 0.50 | 2.41 |
| simulidae | 44.00 | 89.09 | 0.02 | 11.95 | 0.07 | 1.00 | 0.98 | 9.62 |
| staphylinidae | 3.00 | 13.22 | 0.08 | 3.92 | 0.54 | 0.86 | 0.40 | 2.97 |
| tabanidae | 3.00 | 7.14 | 0.02 | 8.00 | 0.08 | 1.00 | 0.90 | 10.44 |
| tipulidae | 9.00 | 11.79 | 0.32 | 1.22 | 0.10 | 0.66 | 0.48 | 2.69 |
| tricladida | 5.00 | 3.82 | 0.16 | 1.21 | 0.10 | 0.80 | 0.30 | 1.70 |
| veliidae | 3.00 | 2.73 | 0.28 | 1.06 | 0.10 | 0.44 | 0.16 | 1.71 |
| **(c) Coarse sand** |  |  |  |  |  |  |  |  |
| aeshnidae | 5.00 | 4.50 | 0.06 | 2.19 | 39.30 | 0.96 | 0.66 | 3.95 |
| aeglidae | 6.00 | 10.98 | 0.02 | 5.87 | 68.30 | 0.74 | 0.72 | 5.53 |
| ancylidae | 4.00 | 17.61 | 0.02 | 7.52 | 29.30 | 0.98 | 0.72 | 7.74 |
| baetidae | 73.00 | 45.37 | 0.02 | 6.58 | 54.10 | 1.00 | 1.00 | 7.75 |
| belostomatidae | 8.00 | 17.37 | 0.02 | 6.86 | 68.30 | 0.74 | 0.84 | 6.53 |
| bivalvia | 6.00 | 13.61 | 0.02 | 5.28 | 68.30 | 0.88 | 0.72 | 6.94 |
| caenidae | 35.00 | 54.97 | 0.02 | 8.11 | 68.30 | 0.86 | 1.00 | 7.18 |
| calamoceratidae | 26.00 | 16.28 | 0.10 | 1.67 | 68.30 | 0.76 | 0.78 | 3.76 |
| calopterygidae | 39.00 | 17.80 | 0.06 | 2.17 | 58.40 | 0.82 | 0.78 | 3.26 |
| ceratopogonidae | 17.00 | 12.51 | 0.02 | 2.30 | 48.30 | 1.00 | 0.96 | 3.76 |
| chironomidae | 142.00 | 54.41 | 0.06 | 1.71 | 63.80 | 0.80 | 0.88 | 3.28 |
| coenagrionidae | 12.00 | 9.66 | 0.06 | 2.65 | 68.30 | 0.62 | 0.72 | 2.86 |
| collembola | 25.00 | 23.06 | 0.02 | 5.01 | 63.80 | 0.68 | 0.96 | 6.48 |
| corixidae | 6.00 | 5.02 | 0.10 | 2.22 | 58.40 | 0.96 | 0.66 | 3.60 |
| culicidae | 10.00 | 14.32 | 0.02 | 5.39 | 63.80 | 1.00 | 1.00 | 5.65 |
| dytiscidae | 6.00 | 28.73 | 0.02 | 13.77 | 68.30 | 0.98 | 0.84 | 12.34 |
| elmidae | 41.00 | 61.76 | 0.02 | 4.75 | 68.30 | 0.74 | 0.98 | 5.69 |
| gerridae | 6.00 | 4.80 | 0.12 | 1.99 | 36.45 | 0.64 | 0.40 | 2.31 |
| gyrinidae | 25.00 | 16.18 | 0.02 | 4.37 | 40.07 | 0.90 | 0.96 | 5.85 |
| hebridae | 5.00 | 5.79 | 0.12 | 1.33 | 63.80 | 0.52 | 0.50 | 2.58 |
| hirudinidae | 70.00 | 42.29 | 0.02 | 5.68 | 32.27 | 1.00 | 1.00 | 5.70 |
| hyalellidae | 7.00 | 9.97 | 0.08 | 1.77 | 49.25 | 0.72 | 0.52 | 3.51 |
| hydrophilidae | 4.00 | 5.96 | 0.02 | 4.38 | 54.10 | 0.54 | 0.82 | 6.80 |
| hydropsychidae | 65.00 | 34.98 | 0.04 | 2.00 | 63.80 | 0.92 | 0.72 | 3.07 |
| leptophlebiidae | 15.00 | 48.71 | 0.02 | 8.14 | 68.30 | 0.56 | 0.96 | 10.53 |
| libellulidae | 30.00 | 15.66 | 0.10 | 1.39 | 61.37 | 0.76 | 0.86 | 3.03 |
| mesoveliidae | 3.00 | 2.29 | 0.56 | 0.73 | 40.70 | 0.42 | 0.20 | 1.80 |
| notonectidae | 11.00 | 8.02 | 0.04 | 2.31 | 51.49 | 0.72 | 0.80 | 3.59 |
| oligochaeta | 49.00 | 23.10 | 0.12 | 1.77 | 63.80 | 0.62 | 0.74 | 2.94 |
| ostracoda | 3.00 | 2.68 | 0.28 | 1.05 | 52.80 | 0.86 | 0.36 | 2.56 |
| philopotamidae | 4.00 | 4.35 | 0.06 | 3.00 | 51.49 | 0.98 | 0.50 | 2.74 |
| philosciidae | 3.00 | 2.56 | 0.38 | 0.97 | 40.70 | 0.62 | 0.18 | 1.88 |
| physidae | 19.00 | 16.12 | 0.02 | 3.80 | 32.00 | 0.96 | 0.94 | 4.63 |
| planorbidae | 12.00 | 8.57 | 0.06 | 2.02 | 68.30 | 0.76 | 0.50 | 2.59 |
| simulidae | 44.00 | 21.16 | 0.04 | 2.42 | 51.49 | 0.88 | 0.92 | 5.05 |
| staphylinidae | 3.00 | 4.55 | 0.26 | 1.76 | 63.80 | 0.62 | 0.50 | 4.84 |
| tabanidae | 3.00 | 3.45 | 0.08 | 2.31 | 36.88 | 0.84 | 0.20 | 1.98 |
| tipulidae | 9.00 | 6.62 | 0.08 | 1.77 | 46.05 | 0.68 | 0.66 | 2.86 |
| tricladida | 5.00 | 10.07 | 0.10 | 2.28 | 68.30 | 0.62 | 0.50 | 2.54 |
| veliidae | 3.00 | 15.85 | 0.04 | 2.82 | 29.30 | 0.90 | 0.52 | 4.01 |
| **(d) Pebble** |  |  |  |  |  |  |  |  |
| aeshnidae | 5.00 | 15.59 | 0.04 | 3.67 | 24.20 | 0.64 | 0.86 | 5.58 |
| aeglidae | 6.00 | 11.65 | 0.12 | 2.52 | 24.20 | 0.54 | 0.70 | 4.02 |
| ancylidae | 4.00 | 6.59 | 0.08 | 2.58 | 3.50 | 0.62 | 0.58 | 3.47 |
| baetidae | 73.00 | 32.22 | 0.08 | 2.02 | 23.35 | 0.58 | 0.92 | 3.21 |
| belostomatidae | 8.00 | 4.66 | 0.24 | 0.89 | 10.01 | 0.68 | 0.52 | 2.60 |
| bivalvia | 6.00 | 5.39 | 0.04 | 2.14 | 3.50 | 0.98 | 0.60 | 2.55 |
| caenidae | 35.00 | 17.99 | 0.04 | 2.75 | 20.40 | 0.76 | 0.88 | 3.95 |
| calamoceratidae | 26.00 | 14.53 | 0.02 | 3.04 | 24.20 | 0.50 | 0.94 | 3.95 |
| calopterygidae | 39.00 | 34.10 | 0.02 | 3.39 | 14.70 | 0.84 | 0.68 | 3.25 |
| ceratopogonidae | 17.00 | 10.86 | 0.06 | 1.31 | 9.92 | 0.60 | 0.56 | 2.26 |
| chironomidae | 142.00 | 86.86 | 0.02 | 5.40 | 20.40 | 0.88 | 0.96 | 3.85 |
| coenagrionidae | 12.00 | 13.81 | 0.06 | 3.96 | 4.04 | 0.92 | 0.74 | 3.94 |
| collembola | 25.00 | 13.74 | 0.02 | 3.26 | 4.40 | 0.76 | 0.94 | 4.39 |
| corixidae | 6.00 | 5.45 | 0.06 | 1.62 | 7.67 | 0.84 | 0.48 | 2.47 |
| culicidae | 10.00 | 7.55 | 0.04 | 2.30 | 10.10 | 0.94 | 0.82 | 3.28 |
| dytiscidae | 6.00 | 4.44 | 0.16 | 1.68 | 20.40 | 0.64 | 0.64 | 3.63 |
| elmidae | 41.00 | 17.13 | 0.12 | 1.59 | 23.35 | 0.72 | 0.82 | 3.52 |
| gerridae | 6.00 | 3.82 | 0.54 | 0.14 | 9.81 | 0.54 | 0.44 | 2.24 |
| gyrinidae | 25.00 | 31.05 | 0.02 | 11.02 | 0.63 | 1.00 | 1.00 | 10.92 |
| hebridae | 5.00 | 4.29 | 0.16 | 1.87 | 4.68 | 0.82 | 0.48 | 2.74 |
| hirudinidae | 70.00 | 33.80 | 0.08 | 2.11 | 13.67 | 0.76 | 0.90 | 4.36 |
| hyalellidae | 7.00 | 6.93 | 0.02 | 2.67 | 4.04 | 0.94 | 0.72 | 3.14 |
| hydrophilidae | 4.00 | 14.29 | 0.10 | 2.74 | 2.60 | 0.86 | 0.60 | 3.20 |
| hydropsychidae | 65.00 | 30.39 | 0.16 | 1.17 | 9.92 | 0.58 | 0.62 | 2.52 |
| leptophlebiidae | 15.00 | 10.48 | 0.02 | 3.08 | 2.90 | 0.94 | 0.82 | 4.17 |
| libellulidae | 30.00 | 14.21 | 0.04 | 2.42 | 7.67 | 0.76 | 0.74 | 2.74 |
| mesoveliidae | 3.00 | 5.64 | 0.18 | 2.27 | 14.70 | 0.76 | 0.30 | 2.06 |
| notonectidae | 11.00 | 7.80 | 0.04 | 1.67 | 4.40 | 0.58 | 0.62 | 2.50 |
| oligochaeta | 49.00 | 38.59 | 0.06 | 2.00 | 10.10 | 0.66 | 0.62 | 3.00 |
| ostracoda | 3.00 | 7.09 | 0.02 | 10.19 | 12.40 | 0.94 | 0.60 | 5.63 |
| philopotamidae | 4.00 | 8.10 | 0.12 | 2.62 | 20.40 | 0.60 | 0.36 | 2.52 |
| philosciidae | 3.00 | 4.70 | 0.10 | 2.66 | 9.70 | 0.74 | 0.62 | 3.92 |
| physidae | 19.00 | 21.80 | 0.02 | 6.19 | 9.90 | 0.96 | 1.00 | 6.82 |
| planorbidae | 12.00 | 7.69 | 0.20 | 1.03 | 22.30 | 0.58 | 0.54 | 2.53 |
| simulidae | 44.00 | 33.81 | 0.02 | 3.80 | 12.40 | 0.96 | 1.00 | 4.02 |
| staphylinidae | 3.00 | 6.66 | 0.02 | 2.45 | 10.10 | 0.56 | 0.54 | 4.98 |
| tabanidae | 3.00 | 3.77 | 0.02 | 1.67 | 14.70 | 0.58 | 0.24 | 2.68 |
| tipulidae | 9.00 | 7.06 | 0.12 | 1.37 | 10.10 | 0.42 | 0.62 | 3.30 |
| tricladida | 5.00 | 8.79 | 0.08 | 3.29 | 20.40 | 0.54 | 0.48 | 2.72 |
| veliidae | 3.00 | 4.21 | 0.30 | 1.47 | 3.23 | 0.66 | 0.52 | 3.27 |

**Table SM7.** Results of the Threshold Indicator Taxa Analysis (TITAN) of the response of the phytoplankton’s community by percentage of stone in sediment stream (a), water temperature (b), water dissolved oxygen (c) and percentage of coarse sand in sediment stream (d) along watershed basin. [freq = number of non-zero abundance values; IndVal = indication value of the taxa; obsiv.prob = probability of an equal or larger IndVal from random permutations; zscore = IndVal expressed as a z-score; 95%, quantile thresholds among bootstrap replicates; purity, proportion of the correct response, whether negative (z - ) or positive (z +); reliability, fidelity of taxa to the group, p ≤ 0.05; z.median = median z-score across replicates].

|  | **freq** | **IndVal** | **obsiv.prob** | **zscore** | **95%** | **purity** | **reliability** | **z.median** |
| --- | --- | --- | --- | --- | --- | --- | --- | --- |
| **(a) Stone** |  |  |  |  |  |  |  |  |
| closterium | 23.00 | 58.25 | 0.04 | 3.80 | 3.58 | 0.96 | 0.90 | 3.98 |
| coelastrum | 14.00 | 41.03 | 0.30 | 0.98 | 7.05 | 0.60 | 0.46 | 2.47 |
| cosmarium | 4.00 | 56.25 | 0.02 | 7.34 | 6.79 | 0.80 | 0.62 | 4.87 |
| crucigenia | 4.00 | 20.00 | 0.28 | 1.22 | 3.00 | 0.42 | 0.14 | 1.59 |
| desmodesmus | 19.00 | 52.08 | 0.02 | 2.37 | 6.04 | 0.90 | 0.70 | 3.43 |
| euastrum | 7.00 | 29.45 | 0.18 | 1.53 | 3.21 | 0.90 | 0.44 | 2.50 |
| euglena | 10.00 | 32.35 | 0.22 | 1.29 | 4.05 | 0.74 | 0.50 | 2.70 |
| gyrosigma | 15.00 | 68.18 | 0.02 | 5.24 | 3.10 | 1.00 | 0.98 | 5.64 |
| hariotina | 4.00 | 12.29 | 0.30 | -0.21 | 7.07 | 0.32 | 0.14 | 1.78 |
| kirchneriella | 9.00 | 29.81 | 0.12 | 1.11 | 4.05 | 0.64 | 0.18 | 1.96 |
| komvophoron | 3.00 | 14.10 | 0.52 | 0.66 | 3.20 | 0.56 | 0.20 | 2.16 |
| micrasterias | 3.00 | 23.08 | 0.06 | 2.95 | 7.05 | 0.96 | 0.42 | 3.25 |
| pediastrum | 16.00 | 40.00 | 0.20 | 0.79 | 7.05 | 0.66 | 0.30 | 1.61 |
| scenedesmus | 14.00 | 58.18 | 0.02 | 4.15 | 7.05 | 0.94 | 0.82 | 3.90 |
| spirogyra | 3.00 | 23.08 | 0.10 | 2.44 | 1.80 | 0.82 | 0.30 | 2.29 |
| spirulina | 6.00 | 50.00 | 0.06 | 3.03 | 6.90 | 0.64 | 0.36 | 2.92 |
| staurastrum | 4.00 | 19.05 | 0.36 | 1.00 | 2.96 | 0.42 | 0.12 | 1.37 |
| stauridium | 3.00 | 14.29 | 0.46 | 1.12 | 4.85 | 0.44 | 0.16 | 1.81 |
| surirella | 21.00 | 46.92 | 0.20 | 0.82 | 4.83 | 0.58 | 0.38 | 1.98 |
| tetraedriella | 3.00 | 14.10 | 0.64 | 0.59 | 3.20 | 0.46 | 0.20 | 1.59 |
| tetraedron | 3.00 | 36.36 | 0.10 | 3.31 | 2.90 | 0.78 | 0.40 | 3.48 |
| tetrastrum | 3.00 | 29.63 | 0.16 | 2.44 | 6.68 | 0.74 | 0.48 | 3.87 |
| treubaria | 5.00 | 19.62 | 0.52 | 0.68 | 3.01 | 0.56 | 0.22 | 1.83 |
| tropidoscyphus | 4.00 | 18.39 | 0.36 | 0.82 | 7.05 | 0.70 | 0.16 | 2.10 |
| westella | 5.00 | 52.94 | 0.04 | 3.92 | 2.48 | 0.94 | 0.68 | 4.49 |
| **(b) Temperature** |  |  |  |  |  |  |  |  |
| closterium | 23.00 | 52.01 | 0.16 | 1.20 | 24.59 | 0.72 | 0.46 | 2.07 |
| coelastrum | 14.00 | 40.30 | 0.24 | 1.12 | 23.84 | 0.72 | 0.42 | 2.03 |
| cosmarium | 4.00 | 29.17 | 0.06 | 3.12 | 24.46 | 0.80 | 0.38 | 2.38 |
| crucigenia | 4.00 | 20.00 | 0.24 | 1.40 | 22.29 | 0.74 | 0.20 | 1.91 |
| desmodesmus | 19.00 | 65.71 | 0.06 | 3.38 | 23.38 | 0.98 | 0.86 | 4.46 |
| euastrum | 7.00 | 23.81 | 0.40 | 0.95 | 24.53 | 0.50 | 0.40 | 2.50 |
| euglena | 10.00 | 45.00 | 0.06 | 3.15 | 23.11 | 0.66 | 0.70 | 3.82 |
| gyrosigma | 15.00 | 58.85 | 0.02 | 3.79 | 23.01 | 0.98 | 0.82 | 4.02 |
| hariotina | 4.00 | 15.56 | 0.62 | 0.24 | 24.46 | 0.64 | 0.20 | 2.06 |
| kirchneriella | 9.00 | 24.62 | 0.56 | 0.11 | 24.53 | 0.48 | 0.26 | 1.67 |
| komvophoron | 3.00 | 12.50 | 0.58 | 0.88 | 24.09 | 0.64 | 0.10 | 1.59 |
| micrasterias | 3.00 | 14.29 | 0.44 | 1.16 | 24.46 | 0.78 | 0.20 | 2.06 |
| pediastrum | 16.00 | 46.70 | 0.10 | 2.18 | 24.42 | 0.42 | 0.58 | 2.64 |
| scenedesmus | 14.00 | 76.67 | 0.02 | 7.51 | 23.90 | 1.00 | 0.98 | 6.20 |
| spirogyra | 3.00 | 18.75 | 0.24 | 1.53 | 21.66 | 0.80 | 0.18 | 1.91 |
| spirulina | 6.00 | 19.88 | 0.40 | 0.48 | 23.98 | 0.56 | 0.22 | 1.81 |
| staurastrum | 4.00 | 16.59 | 0.32 | 0.55 | 24.37 | 0.58 | 0.24 | 1.91 |
| stauridium | 3.00 | 24.80 | 0.12 | 2.70 | 23.29 | 0.90 | 0.44 | 3.11 |
| surirella | 21.00 | 60.36 | 0.02 | 4.78 | 23.62 | 1.00 | 0.96 | 4.71 |
| tetraedriella | 3.00 | 14.10 | 0.56 | 0.55 | 24.26 | 0.58 | 0.16 | 2.17 |
| tetraedron | 3.00 | 33.33 | 0.04 | 3.82 | 24.06 | 0.92 | 0.50 | 3.68 |
| tetrastrum | 3.00 | 29.63 | 0.08 | 3.87 | 24.37 | 1.00 | 0.66 | 4.74 |
| treubaria | 5.00 | 40.14 | 0.04 | 3.52 | 24.12 | 0.88 | 0.78 | 4.67 |
| tropidoscyphus | 4.00 | 33.33 | 0.14 | 2.59 | 22.23 | 0.66 | 0.32 | 2.59 |
| westella | 5.00 | 29.41 | 0.04 | 3.13 | 22.96 | 0.96 | 0.54 | 3.17 |
| **(c) Dissolved oxygen** | |  |  |  |  |  |  |  |
| closterium | 23.00 | 47.56 | 0.16 | 0.66 | 76.14 | 0.60 | 0.42 | 1.65 |
| coelastrum | 14.00 | 64.17 | 0.02 | 5.32 | 68.85 | 1.00 | 0.96 | 5.09 |
| cosmarium | 4.00 | 21.90 | 0.26 | 0.80 | 76.14 | 0.74 | 0.20 | 2.13 |
| crucigenia | 4.00 | 22.22 | 0.14 | 1.46 | 69.43 | 0.52 | 0.28 | 2.06 |
| desmodesmus | 19.00 | 51.65 | 0.26 | 1.58 | 72.10 | 0.82 | 0.56 | 2.65 |
| euastrum | 7.00 | 30.49 | 0.16 | 0.96 | 72.80 | 0.66 | 0.40 | 2.43 |
| euglena | 10.00 | 48.48 | 0.14 | 1.76 | 72.10 | 0.70 | 0.58 | 3.26 |
| gyrosigma | 15.00 | 68.18 | 0.02 | 4.41 | 72.49 | 1.00 | 0.98 | 5.14 |
| hariotina | 4.00 | 16.67 | 0.52 | 0.47 | 75.45 | 0.58 | 0.22 | 1.74 |
| kirchneriella | 9.00 | 29.98 | 0.24 | 1.20 | 72.87 | 0.74 | 0.42 | 2.62 |
| komvophoron | 3.00 | 16.67 | 0.24 | 1.04 | 70.15 | 0.82 | 0.18 | 1.96 |
| micrasterias | 3.00 | 36.36 | 0.04 | 6.55 | 66.80 | 0.96 | 0.50 | 3.89 |
| pediastrum | 16.00 | 64.00 | 0.12 | 2.01 | 75.45 | 0.88 | 0.64 | 2.99 |
| scenedesmus | 14.00 | 54.44 | 0.04 | 3.20 | 69.18 | 0.94 | 0.64 | 3.32 |
| spirogyra | 3.00 | 29.63 | 0.06 | 4.76 | 74.89 | 0.94 | 0.82 | 6.14 |
| spirulina | 6.00 | 24.00 | 0.56 | 0.27 | 72.10 | 0.68 | 0.24 | 1.82 |
| staurastrum | 4.00 | 16.59 | 0.30 | 0.73 | 69.13 | 0.46 | 0.16 | 1.78 |
| stauridium | 3.00 | 25.00 | 0.04 | 3.08 | 64.50 | 1.00 | 0.32 | 2.84 |
| surirella | 21.00 | 55.71 | 0.12 | 1.34 | 74.19 | 0.66 | 0.54 | 2.71 |
| tetraedriella | 3.00 | 16.67 | 0.20 | 1.44 | 76.70 | 0.86 | 0.18 | 1.86 |
| tetraedron | 3.00 | 14.29 | 0.36 | 0.13 | 76.92 | 0.44 | 0.16 | 2.44 |
| tetrastrum | 3.00 | 24.80 | 0.14 | 2.46 | 66.80 | 0.90 | 0.42 | 3.04 |
| treubaria | 5.00 | 13.53 | 0.62 | -0.25 | 79.67 | 0.42 | 0.16 | 1.70 |
| tropidoscyphus | 4.00 | 21.90 | 0.14 | 1.81 | 75.45 | 0.58 | 0.36 | 2.03 |
| westella | 5.00 | 30.77 | 0.14 | 2.11 | 75.45 | 0.64 | 0.28 | 2.44 |
| **(d) Coarse sand** |  |  |  |  |  |  |  |  |
| closterium | 23.00 | 46.30 | 0.38 | 0.27 | 22.90 | 0.52 | 0.30 | 1.74 |
| coelastrum | 14.00 | 54.44 | 0.06 | 3.16 | 20.69 | 0.94 | 0.74 | 3.77 |
| cosmarium | 4.00 | 22.22 | 0.10 | 2.00 | 19.62 | 0.74 | 0.26 | 2.19 |
| crucigenia | 4.00 | 21.90 | 0.20 | 1.30 | 22.90 | 0.60 | 0.14 | 1.86 |
| desmodesmus | 19.00 | 51.65 | 0.16 | 1.78 | 19.97 | 0.84 | 0.50 | 2.51 |
| euastrum | 7.00 | 29.45 | 0.16 | 1.55 | 21.80 | 0.78 | 0.40 | 2.07 |
| euglena | 10.00 | 61.54 | 0.04 | 3.67 | 17.24 | 0.96 | 0.74 | 3.78 |
| gyrosigma | 15.00 | 51.61 | 0.16 | 1.98 | 22.96 | 0.68 | 0.50 | 2.61 |
| hariotina | 4.00 | 25.00 | 0.10 | 2.35 | 21.80 | 0.88 | 0.34 | 2.57 |
| kirchneriella | 9.00 | 42.30 | 0.04 | 2.97 | 19.70 | 0.94 | 0.72 | 4.53 |
| komvophoron | 3.00 | 25.00 | 0.04 | 3.59 | 14.52 | 0.98 | 0.62 | 4.05 |
| micrasterias | 3.00 | 18.30 | 0.18 | 1.58 | 20.50 | 0.68 | 0.34 | 2.68 |
| pediastrum | 16.00 | 56.87 | 0.02 | 2.84 | 20.20 | 0.88 | 0.62 | 3.53 |
| scenedesmus | 14.00 | 73.53 | 0.02 | 5.23 | 18.79 | 0.98 | 0.90 | 4.62 |
| spirogyra | 3.00 | 29.63 | 0.08 | 3.87 | 21.80 | 0.78 | 0.46 | 2.62 |
| spirulina | 6.00 | 30.40 | 0.06 | 2.71 | 20.15 | 1.00 | 0.64 | 3.70 |
| staurastrum | 4.00 | 20.00 | 0.20 | 1.42 | 22.90 | 0.68 | 0.10 | 1.94 |
| stauridium | 3.00 | 13.64 | 0.46 | 0.69 | 20.20 | 0.52 | 0.12 | 1.51 |
| surirella | 21.00 | 64.00 | 0.02 | 4.32 | 20.92 | 0.88 | 0.66 | 2.76 |
| tetraedriella | 3.00 | 21.15 | 0.22 | 1.65 | 23.00 | 0.56 | 0.34 | 2.72 |
| tetraedron | 3.00 | 15.79 | 0.36 | 1.06 | 20.20 | 0.72 | 0.06 | 1.54 |
| tetrastrum | 3.00 | 15.79 | 0.30 | 1.17 | 19.31 | 0.74 | 0.10 | 2.11 |
| treubaria | 5.00 | 22.50 | 0.24 | 1.62 | 21.05 | 0.60 | 0.46 | 2.96 |
| tropidoscyphus | 4.00 | 7.47 | 1.02 | -0.53 | 23.00 | 0.36 | 0.12 | 1.92 |
| westella | 5.00 | 16.16 | 0.62 | 0.22 | 21.80 | 0.50 | 0.16 | 1.42 |

**Table SM8.** Spatial distribution of the adapted Tropical Water Health Index (TWHI) across 30 georeferenced sampling sites based on cost and efficiency indicators.

| Site code | Lat | Lon | TWHI | Class |
| --- | --- | --- | --- | --- |
| 1 | -27.084116 | -52.669447 | 0.5 | Fair |
| 2 | -27.079319 | -52.660619 | 0.47 | Fair |
| 3 | -27.077 | -52.647889 | 0.37 | Poor |
| 4 | -27.069111 | -52.645833 | 0.54 | Fair |
| 5 | -27.055833 | -52.6435 | 0.46 | Fair |
| 6 | -27.063472 | -52.6485 | 0.5 | Fair |
| 7 | -27.006444 | -52.660139 | 0.48 | Fair |
| 8 | -27.030694 | -52.66425 | 0.48 | Fair |
| 9 | -27.056889 | -52.663361 | 0.6 | Good |
| 10 | -27.061361 | -52.666194 | 0.52 | Fair |
| 11 | -27.041806 | -52.668778 | 0.54 | Fair |
| 12 | -27.039611 | -52.650167 | 0.59 | Fair |
| 13 | -27.038417 | -52.64575 | 0.59 | Fair |
| 14 | -27.027833 | -52.633222 | 0.61 | Good |
| 15 | -26.991361 | -52.652361 | 0.52 | Fair |
| 16 | -26.991417 | -52.652389 | 0.5 | Fair |
| 17 | -26.999611 | -52.614556 | 0.44 | Fair |
| 18 | -27.00475 | -52.61675 | 0.55 | Fair |
| 19 | -27.027167 | -52.617944 | 0.54 | Fair |
| 20 | -27.049306 | -52.624667 | 0.63 | Good |
| 21 | -27.118722 | -52.668333 | 0.39 | Poor |
| 22 | -27.121444 | -52.685083 | 0.57 | Fair |
| 23 | -27.111444 | -52.660472 | 0.46 | Fair |
| 24 | -27.095889 | -52.656806 | 0.4 | Poor |
| 25 | -27.095861 | -52.657278 | 0.53 | Fair |
| 26 | -27.09419 | -52.658806 | 0.49 | Fair |
| 27 | -27.092639 | -52.655333 | 0.57 | Fair |
| 28 | -27.027846 | -52.645552 | 0.56 | Fair |
| 29 | -27.090415 | -52.644533 | 0.47 | Fair |
| 30 | -27.089614 | -52.666751 | 0.54 | Fair |

**Script supplementary material 9**. Automated computation of the Tropical Water Health Index (TWHI) using fuzzy logic and PCR aggregation

This R script computes the Tropical Water Health Index (TWHI) based on the Pressure-Condition-Response (PCR) framework.

It performs automatic threshold generation (Q5/Q25/Q50/Q75/Q95) for each indicator, applies trapezoidal fuzzy membership functions, and aggregates standardized scores into harmonized subindices (Pressure, Condition, Response) and the final TWHI classification.

All procedures are reproducible and require no manual editing of thresholds unless a “thresholds” sheet is provided in the Excel input file.

Outputs (CSV):

twhi_out/TWHI_resultados.csv - Final index results (TWHI and class per site)

twhi_out/TWHI_harmonias_por_indicador.csv - Fuzzy harmony scores per indicator

twhi_out/TWHI_thresholds_usados.csv - Thresholds used or generated

# =============================================================

# Script S1 – Tropical Water Health Index (TWHI) computation

# =============================================================

# --- Package setup ------------------------------------------------------------

suppressPackageStartupMessages({

libs <- c("readxl", "dplyr", "tidyr", "janitor", "data.table", "stringr", "purrr")

need <- libs[!sapply(libs, requireNamespace, quietly = TRUE)]

if (length(need)) install.packages(need)

lapply(libs, library, character.only = TRUE)

})

`%||%` <- function(a, b) if (!is.null(a)) a else b

# --- Parameters ---------------------------------------------------------------

excel_path <- "Custos vs Eficiencia.xlsx"

sheet_thr <- NA_character_ # If thresholds sheet exists, it will override auto-generation

id_cols <- c("site_code", "date", "lat", "lon", "id", "site", "code")

# --- Fuzzy membership functions -----------------------------------------------

# Trapezoidal functions applied to standardize indicators to [0,1]

hd_eff <- function(x, a,b,c,d,e) { # Efficiency indicators (higher = better)

y <- numeric(length(x))

y[x <= a | x >= e] <- 0

i <- x > a & x <= b; y[i] <- (x[i]-a)/(b-a)*0.5

i <- x > b & x <= c; y[i] <- 0.5 + (x[i]-b)/(c-b)*0.5

i <- x > c & x <= d; y[i] <- 1 - (x[i]-c)/(d-c)*0.5

i <- x > d & x < e; y[i] <- 0.5 - (x[i]-d)/(e-d)*0.5

y

}

hd_cost <- function(x, a,b,c,d,e) 1 - hd_eff(x, a,b,c,d,e) # Cost indicators (higher = worse)

hd_u <- function(x, a,b,c,d,e, x_best = c) { # U-shaped indicators (optimum ≈ c)

dist <- abs(x - x_best)

hd_cost(dist, a,b,c,d,e)

}

scu_to_hd <- function(class_use) { # Legal compliance (SCU) mapping

m <- c(`0`=1.0, `1`=0.8, `2`=0.6, `3`=0.4, `4`=0.2)

v <- unname(m[as.character(class_use)])

ifelse(is.na(v), NA_real_, v)

}

# --- Data import ---------------------------------------------------------------

stopifnot(file.exists(excel_path))

sheets <- readxl::excel_sheets(excel_path)

read_clean <- function(sh) readxl::read_excel(excel_path, sheet = sh) |> janitor::clean_names()

df_custo <- if ("Custo" %in% sheets) read_clean("Custo") else NULL

df_ef <- if ("Eficiente" %in% sheets) read_clean("Eficiente") else NULL

if (is.null(df_custo) && is.null(df_ef))

stop("At least one sheet ('Custo' or 'Eficiente') must be provided.")

# --- ID alignment --------------------------------------------------------------

add_missing_ids <- function(df, id_pool) {

if (is.null(df)) return(NULL)

for (id in tolower(id_pool)) if (!id %in% names(df)) df[[id]] <- NA

df

}

df_custo <- add_missing_ids(df_custo, id_cols)

df_ef <- add_missing_ids(df_ef, id_cols)

ids_use <- id_cols[id_cols %in% (names(df_custo) %||% character()) |

id_cols %in% (names(df_ef) %||% character())]

if (!length(ids_use)) {

df_custo$id <- seq_len(nrow(df_custo %||% data.frame()))

df_ef$id <- seq_len(nrow(df_ef %||% data.frame()))

ids_use <- "id"

}

# --- Numeric indicators --------------------------------------------------------

num_cols <- function(df, ids) if (is.null(df)) character(0)

else names(df)[sapply(df, is.numeric) & !(names(df) %in% tolower(ids))]

num_custo <- num_cols(df_custo, ids_use)

num_ef <- num_cols(df_ef, ids_use)

# --- Combine datasets ----------------------------------------------------------

join_by_ids <- function(a, b, ids) {

if (is.null(a)) return(b)

if (is.null(b)) return(a)

suppressMessages(full_join(a, b, by = ids))

}

meta <- join_by_ids(

if (!is.null(df_custo)) df_custo[, ids_use, drop = FALSE],

if (!is.null(df_ef)) df_ef[, ids_use, drop = FALSE],

ids_use

)

Xc <- if (!is.null(df_custo)) df_custo[, num_custo, drop = FALSE] else NULL

Xe <- if (!is.null(df_ef)) df_ef[, num_ef, drop = FALSE] else NULL

df <- cbind(meta, Xc, Xe)

# --- Threshold definition ------------------------------------------------------

if (is.na(sheet_thr)) {

all_num <- names(df)[sapply(df, is.numeric) & !(names(df) %in% ids_use)]

if (!length(all_num)) stop("No numeric indicators found.")

origin <- tibble::tibble(

indicator = all_num,

source = case_when(

indicator %in% num_custo ~ "Custo",

indicator %in% num_ef ~ "Eficiente",

TRUE ~ "Desconhecida"

),

layer = ifelse(source == "Custo", "P", "C"),

type = ifelse(source == "Custo", "cost", "eff"),

weight = 1

)

qfun <- function(v) {

v <- suppressWarnings(as.numeric(v)); v <- v[is.finite(v)]

if (!length(v)) return(rep(NA_real_,5))

as.numeric(quantile(v, probs = c(0.05,0.25,0.50,0.75,0.95), na.rm = TRUE, type = 7))

}

quants <- do.call(rbind, lapply(all_num, function(nm) qfun(df[[nm]])))

colnames(quants) <- c("a","b","c","d","e")

thr_tbl <- cbind(indicator = all_num, origin[,c("layer","type","weight")], as.data.frame(quants))

thr_tbl$x_best <- NA_real_

} else {

thr_tbl <- readxl::read_excel(excel_path, sheet = sheet_thr) |> janitor::clean_names()

thr_tbl <- thr_tbl |> mutate(layer = toupper(layer), type = tolower(type))

}

dir.create("twhi_out", showWarnings = FALSE)

data.table::fwrite(thr_tbl, "twhi_out/TWHI_thresholds_usados.csv")

# --- Fuzzy harmonization per indicator -----------------------------------------

compute_hd <- function(df, thr_tbl, ids) {

out <- df

for (k in seq_len(nrow(thr_tbl))) {

ind <- thr_tbl$indicator[k]

if (!ind %in% names(out)) { warning("Missing indicator: ", ind); next }

typ <- thr_tbl$type[k]

a <- thr_tbl$a[k]; b <- thr_tbl$b[k]; c <- thr_tbl$c[k]

d <- thr_tbl$d[k]; e <- thr_tbl$e[k]; xb <- thr_tbl$x_best[k] %||% c

x <- suppressWarnings(as.numeric(out[[ind]]))

hd <- switch(typ,

"eff" = hd_eff(x, a,b,c,d,e),

"cost" = hd_cost(x, a,b,c,d,e),

"u" = hd_u(x, a,b,c,d,e, xb),

"class" = scu_to_hd(x),

{ warning("Unknown type for ", ind, ": ", typ); NA_real_ })

out[[paste0("HD_", ind)]] <- hd

}

cbind(out[, ids, drop = FALSE], out[, grep("^HD_", names(out), value = TRUE), drop = FALSE])

}

hd_df <- compute_hd(df, thr_tbl, ids_use)

data.table::fwrite(hd_df, "twhi_out/TWHI_harmonias_por_indicador.csv")

# --- PCR aggregation -----------------------------------------------------------

aggregate_layers <- function(hd_df, thr_tbl, ids) {

meta <- thr_tbl %>% select(indicator, layer, weight)

long <- hd_df %>%

pivot_longer(cols = starts_with("HD_"), names_to = "HD_name", values_to = "HD") %>%

mutate(indicator = str_remove(HD_name, "^HD_")) %>%

left_join(meta, by = "indicator") %>%

mutate(weight = ifelse(is.na(weight), 1, weight))

subidx <- long %>%

group_by(across(all_of(ids)), layer) %>%

summarise(layer_score = if (all(is.na(HD))) NA_real_

else sum(HD * weight, na.rm = TRUE) / sum(weight[!is.na(HD)]),

.groups = "drop") %>%

pivot_wider(names_from = layer, values_from = layer_score)

for (nm in c("P","C","R")) if (!nm %in% names(subidx)) subidx[[nm]] <- NA_real_

subidx %>%

mutate(

TWHI = rowMeans(select(., P, C, R), na.rm = TRUE),

Class = cut(TWHI,

breaks = c(-Inf, 0.2, 0.4, 0.6, 0.8, Inf),

labels = c("Crítico","Ruim","Moderado","Bom","Excelente"),

right = TRUE, ordered_result = TRUE)

)

}

res_layer <- aggregate_layers(hd_df, thr_tbl, ids_use)

out <- df %>% left_join(res_layer, by = ids_use)

data.table::fwrite(out, "twhi_out/TWHI_resultados.csv")

message("✓ TWHI computation completed.",

"\n - twhi_out/TWHI_thresholds_usados.csv",

"\n - twhi_out/TWHI_harmonias_por_indicador.csv",

"\n - twhi_out/TWHI_resultados.csv")
